# Parity and Longevity of *Aedes aegypti* According to Temperatures in Controlled Conditions and Consequences on Dengue Transmission Risks

**DOI:** 10.1371/journal.pone.0135489

**Published:** 2015-08-10

**Authors:** Daniella Goindin, Christelle Delannay, Cédric Ramdini, Joël Gustave, Florence Fouque

**Affiliations:** 1 Laboratory of Medical Entomology, Unit Environment and Health, Institut Pasteur de la Guadeloupe, 97183 Les Abymes, Guadeloupe; 2 Service de Lutte Anti-Vectorielle, Agence Régionale de Santé, Dothémare, Les Abymes, Guadeloupe; 3 Vector Environment and Society Unit, Special Programme for Research and Training in Tropical Diseases (TDR), World Health Organization, 20, Avenue Appia, CH-1211 Geneva 27, Switzerland; Metabiota, UNITED STATES

## Abstract

**Background:**

In Guadeloupe, *Aedes aegypti* mosquitoes are the only vectors of dengue and chikungunya viruses. For both diseases, vector control is the only tool for preventing epidemics since no vaccine or specific treatment is available. However, to efficiently implement control of mosquitoes vectors, a reliable estimation of the transmission risks is necessary. To become infective an *Ae*. *aegypti* female must ingest the virus during a blood meal and will not be able to transmit the virus during another blood-meal until the extrinsic incubation period is completed. Consequently the aged females will carry more infectious risks. The objectives of the present study were to estimate under controlled conditions the expectation of infective life for females and thus the transmission risks in relation with their reproductive cycle and parity status.

**Methodology/Principal Findings:**

Larvae of *Ae*. *aegypti* were collected in central Guadeloupe and breed under laboratory conditions until adult emergence. The experiments were performed at constant temperatures (± 1.5°C) of 24°C, 27°C and 30°C on adults females from first generation (F1). Females were kept and fed individually and records of blood-feeding, egg-laying and survival were done daily. Some females were dissected at different physiological stages to observe the ovaries development. The data were analyzed to follow the evolution of parity rates, the number of gonotrophic cycles, the fecundity and to study the mean expectation of life and the mean expectation of infective life for *Ae*. *aegypti* females according to temperatures. The expectation of life varies with the parity rates and according to the temperatures, with durations from about 10 days at low parity rates at the higher temperature to an optimal duration of about 35 days when 70% of females are parous at 27°C. Infective life expectancy was found highly variable in the lower parous rates and again the optimal durations were found when more than 50% of females are parous for the mean temperatures of 27°C and 30°C.

**Conclusion:**

Parity rates can be determined for field collected females and could be a good proxy of the expectation of infective life according to temperatures. However, for the same parity rates, the estimation of infective life expectation is very different between *Ae*. *aegypti* and *Anopheles gambiae* mosquitoes. Correlation of field parity rates with transmission risks requires absolutely to be based on *Ae*. *aegypti* models, since available *Anopheles sp*. models underestimate greatly the females longevity.

## Introduction

In Guadeloupe, the mosquito *Aedes aegypti* is the vector of Dengue Viruses (DENV) and more recently Chikungunya Virus (CHIKV). Dengue burden is increasing all over the Americas [1, 2]. In the French West Indies island of Guadeloupe, DENV epidemics have been occurring at shorter time intervals since the last 20 years [3] with an increasing number of cases, reaching 45,000 cases i.e. 1/10 of the population, during the last epidemic of 2009–2011 [4, 5]. *Ae*. *aegypti* is recognized as the only DENV vector [6]. Two years later, at the end of 2013, CHIKV was introduced in the Island of Saint-Martin [7], it was subsequently transported to the other French territories in Guadeloupe and Martinique islands where major epidemics are now reported with about 81,200 and 72,200 clinical cases reported respectively, between December 2013 and December 2014 [8]. Furthermore, CHIKV is now spreading in all the Caribbean and the tropical American countries [9]. *Ae*. *aegypti* mosquitoes are the only CHIK vectors in most islands. Since no vaccine or specific treatment is available for both diseases, the prevention of epidemics relies on vector control. The Health Authorities are thus desperately looking for both better entomological indicators to estimate the epidemic risks and vector control options. For this purpose a collaborative study between Guadeloupe, Martinique and French Guiana was implemented to collect entomological indicators in relation with Arboviruses surveillance. Among the indicators tested, the field parity rates of *Ae*. *aegypti* females, that is the percentage of females having already deposited a batch of eggs, were determined to estimate females survival under field conditions [10]. However, the relation between parity rates and the longevity/expectation of life of *Ae*. *aegypti* females has never been studied and a better knowledge of this relationship is needed if we want to use the parity rates as a proxy of female duration of life in field conditions [11].

**Fig 1:**
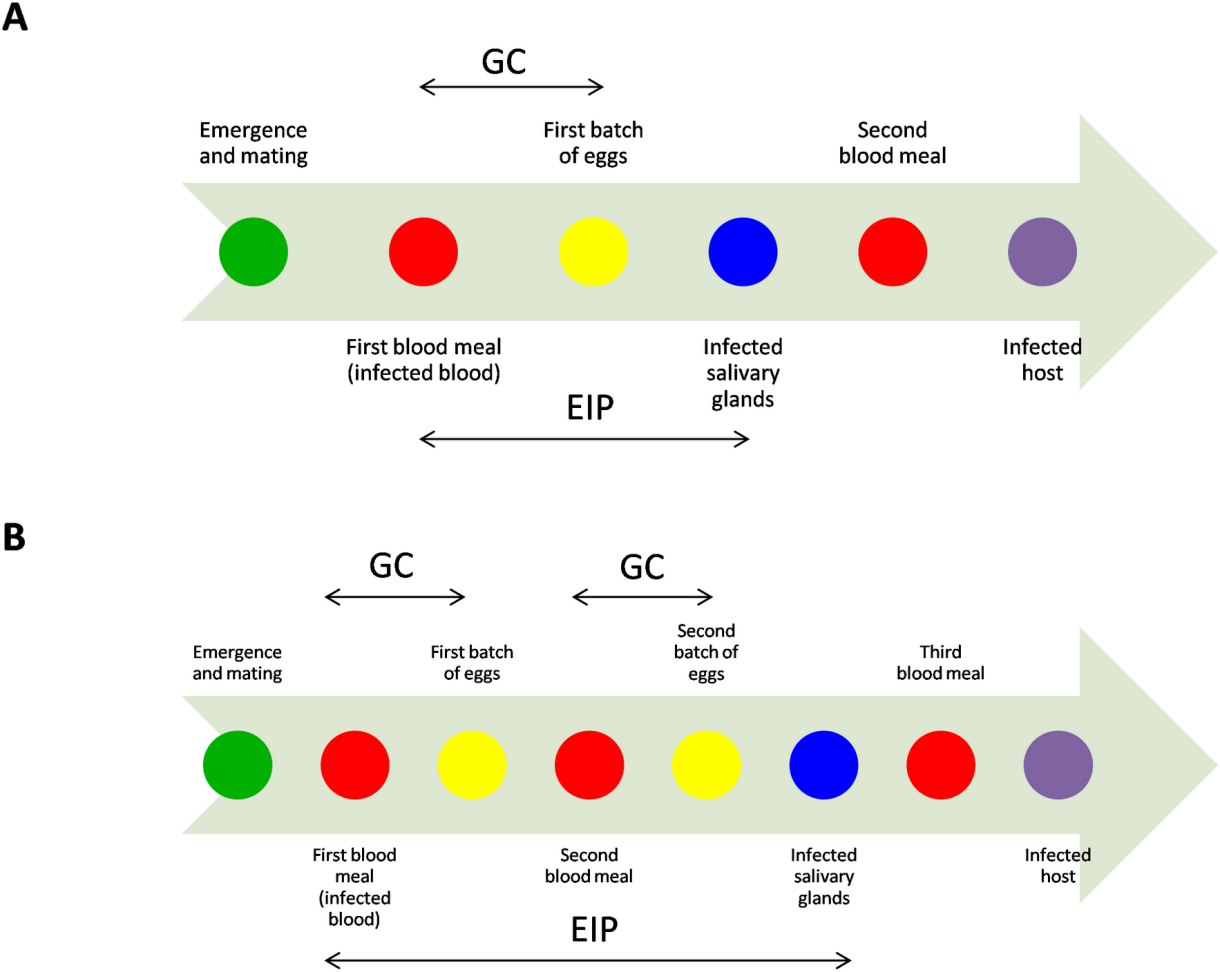
Graphical representation of the biological events occurring in the *Aedes aegypti* female life-cycle at optimal (A) and lower (B) temperatures in relation with the dengue and chikungunya virus infections, amplification and transmission. GC = Gonotrophic Cycle, EIP = Extrinsic Incubation period.

The transmission of DENV and CHIKV to humans by the mosquitoes vectors is achieved through blood feeding [12–14]. In the course of their adult life, the female mosquitoes mates with males after their emergence and start looking for a blood meal to obtain the necessary amino acids for eggs maturation which last a few days [15]. Then the females oviposit in a suitable breeding-site and look for another blood meal, but this pattern is not so strict for *Ae*. *aegypti* mosquitoes that can take multiple blood meals at any time of the egg development [16, 17]. The mosquito reproductive cycle starting at the blood meal and ending with egg-laying, also called the gonotrophic cycle (GC) is continuous during all the female life and is temperature-dependent [18, 19]. During their blood meals, *Ae*. *aegypti* females can ingest the virus that will disseminate into the mosquito body by passing first into the midgut, then crossing the intestinal barriers, amplifying into the haemocoele and eventually reaching the ovaries and the salivary glands. The infective cycle starting when the virus is ingested and ending when the virus reaches the salivary glands is called the extrinsic incubation period (EIP) and is also temperature-dependent [20, 21]. Both cycles (GC and EIP) are thus taking place in the mosquito body simultaneously but with different time durations and different temperature-dependent responses. DENV and CHIKV transmission are more efficient when CG and EIP are shorter and transmission will not occur if the mosquito does not survive long enough (Fig 1). However, for viruses transmitted by *Ae*. *aegypti*, the GC duration is not that critical since females can bite at all times [22]. Nevertheless, the percentage of females that have already deposited their eggs, called the parous females, increases as the age of the mosquito population increases, concomitantly with the transmission risks.

Under field conditions and because EIP duration can last for several days, the expectation of life or survival rate for *Ae*. *aegypti* female is a critical parameter to estimate the mosquito vectorial capacity [23, 24]. Many studies have attempted to estimate this survival directly through capture-mark-release-recapture [25–27] or indirectly with the parity rates assuming a direct relation between parity rates and survival [10, 11, 24]. The first methods although more accurate are not available for routine surveillance. On the contrary, the estimation of parity rates from field collections is more feasible. But, to estimate the survival from the parity rates requires some basic knowledge on the mosquito biology in specific environment. If the relationship between the percentage of parous females and the mean duration of life can be accurately determined, it could help in designing indicators/proxy of the risk of encountering female mosquitoes infected by the dengue/chikungunya viruses.

The objectives of the experiments presented herein were thus to study under controlled conditions the duration and numbers of GC, in relation to the female survival and fecundity at different temperatures. The results were then used to analyze the evolution of the parity rates in relation with the female survival. A function of the daily survival rates estimated from the parity rates according to temperatures was then estimated and used to model the expectation of infective life depending on longevity and EIP duration and according to temperatures. The final goals of these experiments are to provide additional tools to estimate the infective risks for the dengue vector *Aedes aegypti*. The actual estimation of the dengue transmission risks is based on larval and pupae sampling, but these mosquito stages are not the transmitting ones. Thus, an estimation of the risks based on adult information is better related to transmission patterns.

## Materials and Methods

### Mosquito Collection

Mosquitoes were collected in Guadeloupe (French West Indies), in the localities of Baie-Mahault (Lat 16°26”, Long -61°59”) and Petit-Bourg (Lat 16°18”, Long -61°59”), located in Basse-Terre island, during February 2012. These locations were chosen because they are in a central position with mosquito populations submitted to temperatures and rainfalls very close to the mean averages of Guadeloupe. Mosquito larvae and pupae were collected in domestic breeding sites (flower pots, tires, old garbage, old refrigerators and others) found in or around private houses. The field collections were performed during routine survey carried out with the Vector Control Agency of Guadeloupe (Agence Régionale de Santé, French Ministry of Health) and permission to collect mosquito larvae was obtained from the residents. The water with larvae and pupae was filtered with a net and the mosquitoes were collected in small plastic trays with some water from the breeding site. The larvae and pupae were then transported to the insectarium where they were kept under controlled conditions in Bioquip plastic containers filled with de-chlorinated water and fed with yeast. Pupae were placed in Bioquip breeders until their emergence. Adult female mosquitoes from field generation (F0) were then removed from the breeders and kept in cages where they had the opportunity to mate and fed with a solution of 10% sucrose. First generation of laboratory adult females (F1) of 3 to 20 days old were used in the experiments. Before the experiments, mosquitoes were bred at room temperature equivalent to the climatic conditions of Pointe-à-Pitre.

### Experimental study of the gonotrophic cycles, fecundity and survival of females

The duration and number of the gonotrophic cycles, the fecundity *i*.*e*. the number of eggs per batch and the duration of life of female mosquitoes were studied at 3 temperatures: 24°C, 27°C and 30°C, close to the mean lower, mean and mean higher temperatures in Guadeloupe. These three temperatures were chosen to coincide with the temperatures that occur naturally such as in 2011 when temperatures ranged from a minimum of 20°C to a maximum of 32°C, with an average temperature of about 26°C (weather station du Raizet, Les Abymes, climatic data communicated by France Meteo of Guadeloupe). Mosquitoes were reared and maintained at 12:12 light:dark cycle, and 80% ± 10% humidity which are conditions close to the natural ones in Guadeloupe. For the 24°C and 27°C, the room temperature was controlled by an air conditioner (Model: WN 9 R410AW). For the temperature of 30°C, the room was naturally left at 30°C, due to the outside climatic conditions. The insectarium temperatures were monitored electronically with daily record of maximum and minimum with a digital thermometer. The mean standard deviation of temperatures fluctuations was 1.5°C. During the experiments, female mosquitoes were anesthetized with ice and placed individually in small pots closed with tissue nets. Human blood meals for optimal fecundity [28] were offered each female every day until the females abdomen were fully engorged, then again every day after the first batch of eggs was deposited, for the subsequent gonotrophic cycles. Blood-fed females were kept in tubes containing humid filter paper where they could lay their eggs and fed with 10% sucrose solution available in a wad of cotton placed at the top of the tube. Every day after the blood-meal, the filter papers were checked for the presence of eggs and wetted. This procedure took place in the morning between 8 and 10 a.m. When egg laying started, the female mosquito was ice anesthetized daily to remove the blotting paper with the eggs and to place another paper in the small pot. The data on emergence, blood meals, spawning, death and number of eggs were reported daily. In the first experiment, 81 females were followed during 30 days at a mean temperature of 24°C. In the second experiment, 87 females were followed during 25 days at a mean temperature of 27.0°C. In the third experiment, 95 females were followed during 19 days at a mean temperature of 30°C.

### Observation of the ovarian development during the gonotrophic cycle

During all experiments, some females were killed (frozen) and dissected under a binocular microscope to observe ovaries and ovarian tracheoles to better follow the eggs maturation. The dissections were performed to better associate the observation of the ovarian development and the parity status and in order to determine the parity status from ovaries dissection in field collected females. Mosquitoes were placed on a histological slide (Thermo scientific Menzel-Gläser 76mm x 26mm, ground edges 90° frosted end) in a drop of NaCl 0.9% (5 mL Miniversol, lot number 5700, 0459 CE). Legs, wings, head and thorax were eliminated using two needles (BD Vacutainer Precision Glide REF 360213, 21Gx1.5", 0.8x38mm) to keep only the abdomen. The ovaries were removed gently raising the penultimate tergite while pulling down the last tergite. The observation of ovaries and ovarian tracheoles was done under a microscope (Fluorescence microscope Olympus BH2-RFCA). According to the physiological stage, the females of *Ae*. *aegypti* were classified as unfed, blood-engorged, half-gravid and gravid. Only unfed and blood-engorged females could allow the observation of ovaries for the parity status since half-gravid and gravid females ovaries were already full with eggs. The classification of the ovaries according to Detinova [29] was not possible in most cases.

### Data analysis

All data were compiled in tables with Microsoft office Excel software (2007 version). The raw data were transformed to analyze parity, survival, number and duration of gonotrophic cycles (GC), fecundity per cycle and per female. All statistical analysis were performed with Microsoft office Excel software (2007 version). The transformed data were represented graphically as functions of time (in days) for each of the 3 temperatures. Temperature threshold was extrapolated for the reproductive cycle. Analysis of variance one-way statistical test was used to estimate the significance of the relationship between temperatures, durations of GCs, and number of eggs per female. Kruskal-Wallis independence tests were used to analyze the relation between temperature and number of eggs per gonotrophic cycle [30]. Tukey-type tests were used for the multiple comparisons between proportions which included the proportions (%) of females taking a blood-meal, the proportions (%) of parity rates for blood-engorged females, the proportions (%) of overall parity rates and the proportions (%) of survival at the age of 25 days [30]. The Mann-Whitney non-parametric tests were used to compare the durations of the gonotrophic cycles versus the age of the females at the different temperatures. For each temperature, the mean expectation of life (f_i_) of the females was estimated for each day (i) of the experiments as follow:

$$fi=\sum_{i=1}^{n}(di.ai)/Ni$$

with f_i_ = mean expectation of life at day i, d_i_ = number of dead females at day i, a_i_ = age of females in days at day i and N_i_ = total number of females alive at day i, i = 1 to n, n = last day of experiment. The expectation of life was then transform in a daily probability of survival (p_i_) [31]. A mean duration of infective life was estimated using the estimated EIP values for 50% of the female population (EIP_50_) at different temperatures according an exponential regression. The EIP_50_ values used in the regression were 29.60 days, 11.10 days, 5.16 days and 5.16 at constant temperatures of respectively 20°C, 26°C, 30°C and 35°C obtained after experimental infections of *Ae*. *aegypti* females of Thailand [20]. The equation of the regression was:

$${EIP}_{50}=295.43e(0.123.T)\;,\;{\text{r}}^{2}$$

with T = temperature.

The expectation of infective life I_i_ at day i was then extrapolated from the daily probability of survival (p_i_) and the EIP_50,i_ duration according to [31]:

$$Ii=(1/-{\text{log}}_{\;e}(pi))-{EIP}_{50,\;i}$$

Finally the expectation of infective life was plotted against the parity rates obtained for each day of the experiment and each temperature.

## Results

### Evolution of the parity rates

**Fig 2:**
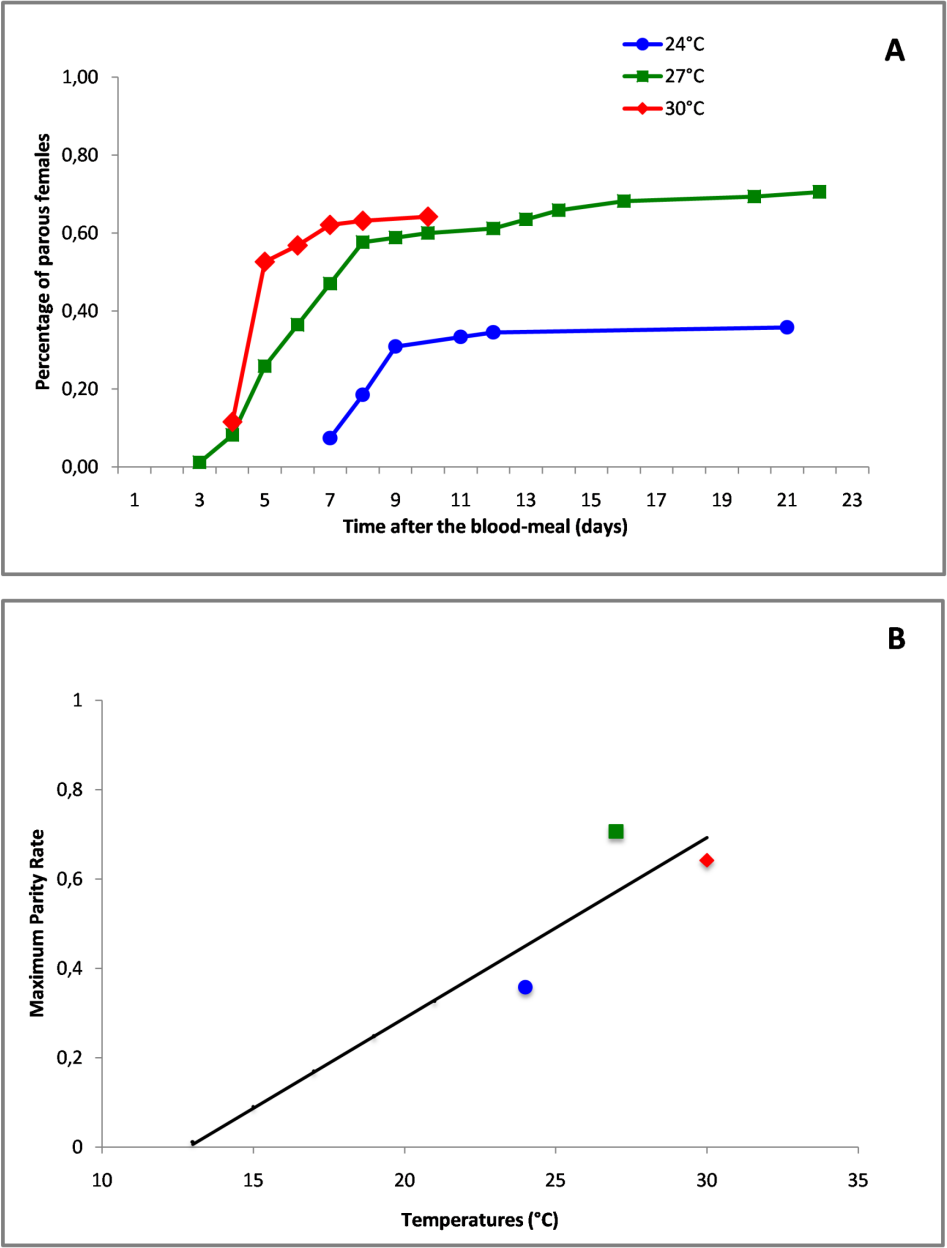
Evolution of the parity rates of *Aedes aegypti* females in time under laboratory conditions at mean constant temperatures of 24°C, 27°C and 30°C. **(A)** Parity rates after the blood meal was taken at day 0. **(B)** Temperature threshold to complete the gonotrophic cycle.

The percentage of females that have accepted to take a blood meal varies according to the temperatures but never reached 100%. Only 49 females (60%), 73 females (86%) and 91 females (96%) accepted the blood-meal at 24°C, 27°C and 30°C respectively, these percentages were found significantly different from each other (Table 1) indicating that temperatures affect the feeding behavior of *Ae*. *aegypti* females (S1 Table). Among the blood-engorged females, only some of them became parous and deposited a batch of eggs. The parity rates of the blood-engorged females varies from 59.2% (29 of 49), 82.2% (60 of 73) and 67% (61 of 91) at 24°C, 27.0°C and 30°C respectively. The high parity rate observed at 27°C was significantly different of the parity rates observed at 24°C and 30°C (Table 1), but no significant difference was found between the parity rates of blood-engorged females at 24°C and 30°C. Temperatures are thus influencing not only the blood meal appetite but also the ability to mature and deposit the eggs, with the lower and high experimental temperatures being less favorable than the medium temperature. The overall parity rates for *Ae*. *aegypti* females varies from 35.8% (29 of 81) to 70.6% (60 of 85) and 64.2% (61 of 95) at 24°C, 27.0°C and 30°C respectively. These proportions were found significantly different between the lowest temperature (24°C) and the highest temperatures (27°C and 30°C) (Table 1), but the difference was not significant between the 2 highest temperatures. Consequently parity rates are temperature-dependent with the most unfavorable temperature being the lower temperature of 24°C and the optimum being 27.0°C. The percentage of parous females according to time was exponential with a different slope according to temperatures (Fig 2A). We observe that the maximum of the parity rate is quickly achieved at the highest temperature of 30°C. The highest parity rate is obtained for the medium temperature of 27°C after 21 day. And finally a poor parity rate is obtained at the lower temperature of 24°C. Consequently, higher temperatures will enhance virus transmission, not only by shortening the EIP but also by optimizing the biting and the parity of female mosquitoes. The variation of the maximum parity rates obtained at the 3 tested temperatures allows to determine the temperature threshold being the lower limit at which the *Ae*. *aegypti* mosquitoes of Guadeloupe will never became parous and thus stop their development (Fig 2B). A linear regression for the tested temperatures showed that the threshold is 12.71°C. Also, the coefficient of regression was not satisfactory due to the small number of temperatures tested; the level of 12°C is realistic since the lowest temperatures observed in Guadeloupe in the mountains area of La Soufrière are around 14°C during the month of January and no *Ae*. *aegypti* are found in this area during the same period.

**Table 1:** Tukey-type test results for comparison among proportion of blood feeding females, parity rate of blood-engorged females, overall parity rates, survival at 25 days of age, proportion of females having 2 GCs and the proportion of females having 3 GCs, at the different experimental temperatures.

Proportions (%) to be compared	Difference	SE	q	q(0.05,∞,3)	Conclusion	
	p'B-p'A				if q > q(0.05,∞,3), then reject H0	
***Blood-feeding***						
30 vs 24*	19.370	1.527	12.682	3.314	*Reject H0*	*p(30) ≠ p(24)*
30 vs 27*	8.245	1.499	5.501	3.314	*Reject H0*	*p(30) ≠ p(27)*
27 vs 24*	11.125	1.559	7.135	3.314	*Reject H0*	*p(27) ≠ p(24)*
***Parity rates of blood-engorged females***						
27 vs 24*	10.715	1.862	5.754	3.314	*Reject H0*	*p(24) ≠ p(27)*
27 vs 30*	7.245	1.587	4.567	3.314	*Reject H0*	*p(27)≠p(30)*
30 vs 24	3.470	1.787	1.942	3.314	Accept H0	p(30) = p(24)
***Overall parity rates***						
27 vs 24*	15.535	1.568	9.907	3.314	*Reject H0*	*p(24) ≠ p(27)*
27 vs 30	2.835	1.508	1.880	3.314	Accept H0	p(27) = p(30)
30 vs 24*	12.700	1.527	8.315	3.314	*Reject H0*	*p(30) ≠ p(24)*
***Survival at 25 days of age***						
27 vs 24*	20.240	1.568	12.908	3.314	*Reject H0*	*p(24) ≠ p(27)*
27 vs 30*	9.735	1.508	6.455	3.314	*Reject H0*	*p(27) ≠ p(30)*
30 vs 24*	10.505	1.527	6.878	3.314	*Reject H0*	*p(30) ≠ p(24)*
***Porportions of females having 2 GCs***						
27 vs 24*	8.195	2.274	3.603	3.314	*Reject H0*	*p(24) ≠ p(27)*
27 vs 30	2.585	1.834	1.409	3.314	Accept H0	p(27) = p(30)
30 vs 24	5.610	2.268	2.473	3.314	Accept H0	p(30) = p(24)
***Porportions of females having 3 GCs***						
27 vs 24*	22.180	2.274	9.752	3.314	*Reject H0*	*p(24) ≠ p(27)*
27 vs 30*	8.310	1.834	4.531	3.314	*Reject H0*	*p(27) ≠ p(30)*
30 vs 24*	13.870	2.268	6.114	3.314	*Reject H0*	*p(30) ≠ p(24)*

* for significant differences

### Duration and number of gonotrophic cycles

**Fig 3:**
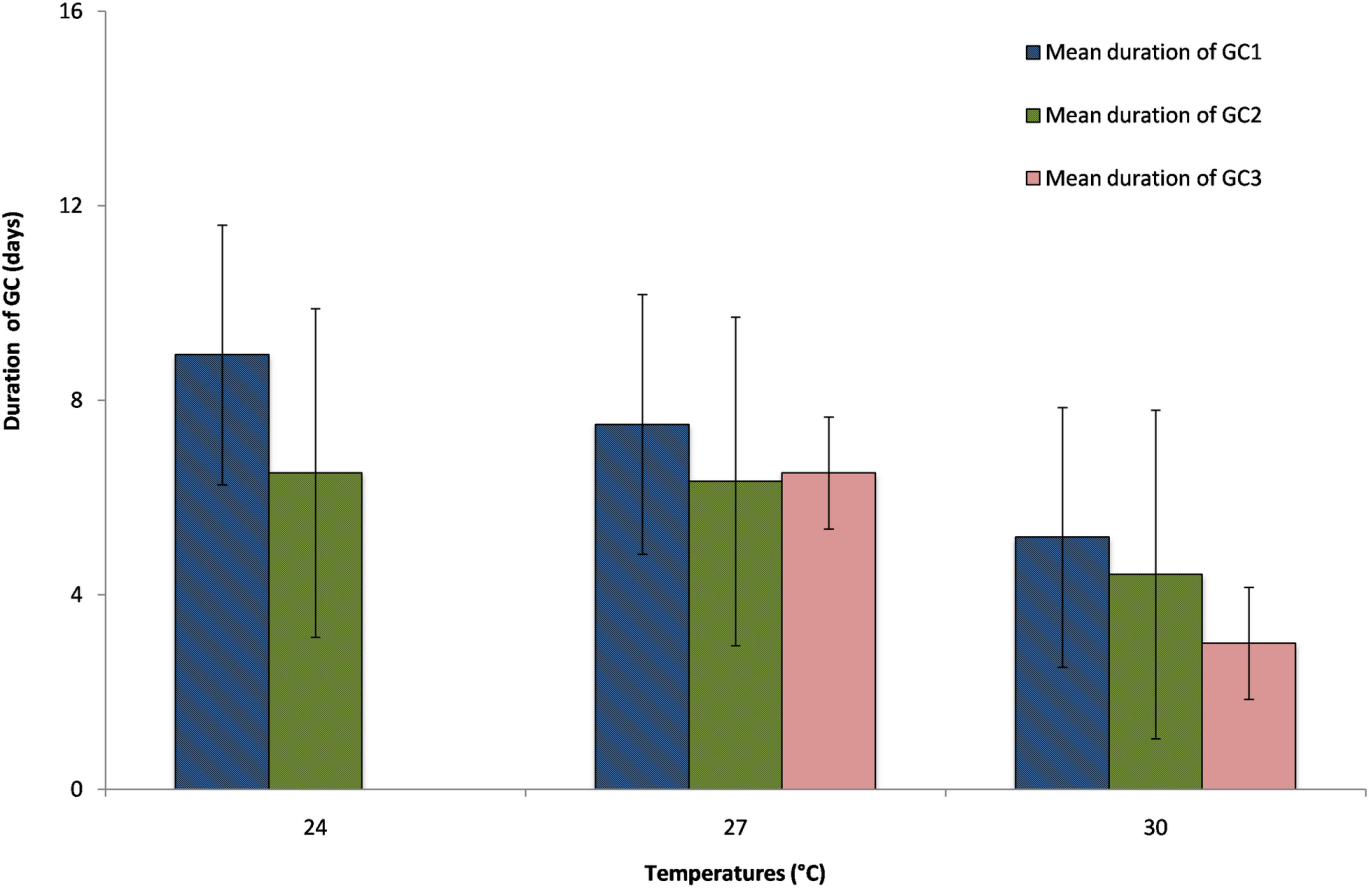
Mean duration and standard errors of the gonotrophic cycles (GCs) at the experimental temperatures of 24°C, 27°C and 30°C. GC1: first gonotrophic cycle, GC2: second gonotrophic cycle, GC3: third gonotrophic cycle.

The duration of the GCs was considered as the number of days between the blood-meal and the first egg laying, even if most females layed eggs for several days during each GC. Also the second blood meal was proposed the day after the first egg-laying, some females continued to deposit eggs resulting from the first CG (GC1) even after their engorgement. The duration of the second GC (GC2) was considered as the number of days between the second blood-meal and the second batch of eggs after an interruption of at least one day. The same procedure was applied for the third and following blood-meals. The duration and the number of GCs varied with temperatures (Fig 3). The longer GC mean duration of 8.41 (± 2.67) days was observed at 24°C, the medium GC mean duration of 7.10 (± 3.38) days was observed at 27°C and the shorter GC mean duration of 4.92 (±1.15) days was observed at 30°C (S2 Table). The time required by *Ae*. *aegypti* females to complete their GC was highly variable according to temperatures with the highest temperature giving the shorter GC durations. The duration of GC1 and GC2 were significantly different according to temperature (S1 File, for GC1, F_2,147_ = 34.60, *P ≤ 0*.*005*; for GC2, F_2,56_ = 6.52, *P ≤ 0*.*005*). The number of GC observed per females varied from 2 GCs at 23°C to 3 GCs at 27°C and 30°C. Nevertheless, not all females were able to bite more than once to develop 2 or 3 distinct GCs regardless of the temperatures. The percentage of females having 2 GCs was 28%, 43%, 39%, and these percentages were significantly different between the lower temperature of 23°C and the medium temperatures of 27°C (Table 1), but no significant difference was found between the 2 highest temperatures and also between 23°C and 30°C. The percentage of females having 3 GCs was 0%, 10%, 3% at 24°C, 27°C and 30°C respectively and the differences were found significant between all temperatures (Table 1). The highest percentages for 2 GCs and 3 GCs were thus found at 27°C. From an epidemiological perspective the shorter duration of the GC and the ability of the *Ae*. *aegypti* female to bite for several GCs will enhance virus transmission [32, 33] and the above results show that if the highest temperature of 30°C is significantly more favorable for shorter GCs, the medium temperature of 27°C allows the female to develop significantly more GCs, and consequently more blood-feeding and more contact with the human host. Furthermore, the duration of the GCs varied not only with temperature, but also with the age of the females since the first GC produced by the youngest females was significantly longer than the following one produced by the older females (Table 2) for all studied temperatures (Fig 3).

**Table 2:** Mann-Whitney non-parametric results for the durations of the gonotrophic cycles versus the age of the females at the different temperatures.

Temperatures	n1	R1	n2	R2	U	U critical	Conclusion				
***24°C***	29	480	8	37	***187***	***164***	*Reject H0*				
							*Duration of GC1 is not the same as duration of GC2*				
***27°C***	60	2579.5	27	1248.5	***870*.*5***	***791*.*3***	*Reject H0*				
							*Duration of GC1 is not the same as duration of GC2*				
***30°C***	61	2222.5	24	1418	***1132*.*5***	***714*.*2***	*Reject H0*				
							*Duration of GC1 is not the same as duration of GC2*				

n1 = the number of females observed for GC1; R1 = rank sum for the durations of GC1; n2 = the number of females observed for GC2; R2 = rank sum for the durations of GC2; U = ((n1*n2)+((n1*(n1+1))/2)-R1); U critical = μ-(1.64*(SD-0.5)), see details on Zar, 1984. H0: There isn't significant differences between the duration of GC1 and GC2. If U < U critical, we can't reject H0. If U > U critical, we can't accept H0.

**Fig 4:**
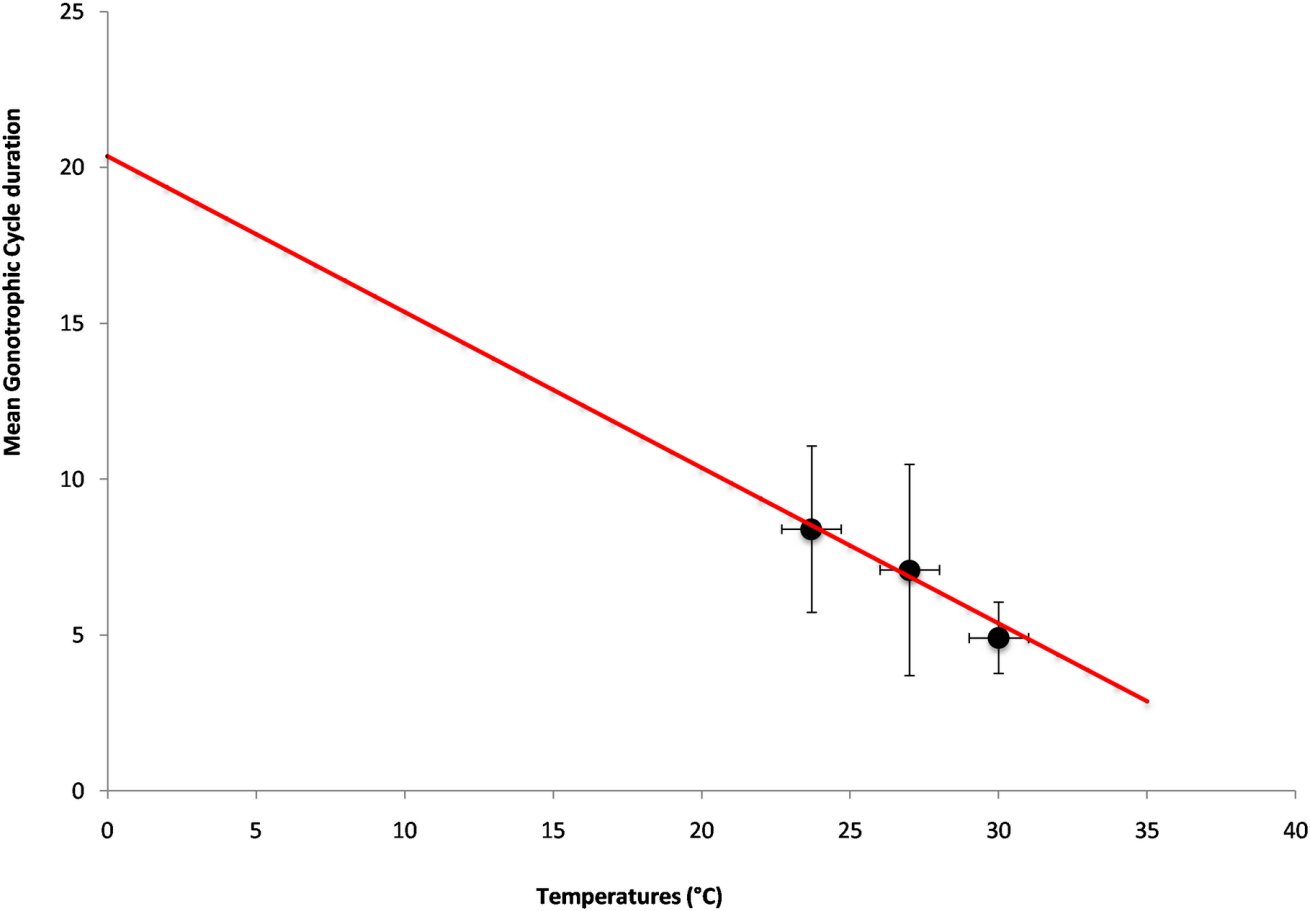
Linear regression representing the mean durations of the Gonotrophic Cycles as a function of temperatures (*y* = −0.4998*x* + 20.368, r^2^ = 0.98).

The data on the CGs durations and numbers coupled with the parity rates show that the force of transmission strongly increase with temperature. The duration of the GCs considered as a function of temperature can be estimated through a linear regression (Fig 4) and show that *Ae*. *aegypti* females CGs are strongly temperature-dependent because the 3 points are almost aligned.

### Fecundity

**Fig 5:**
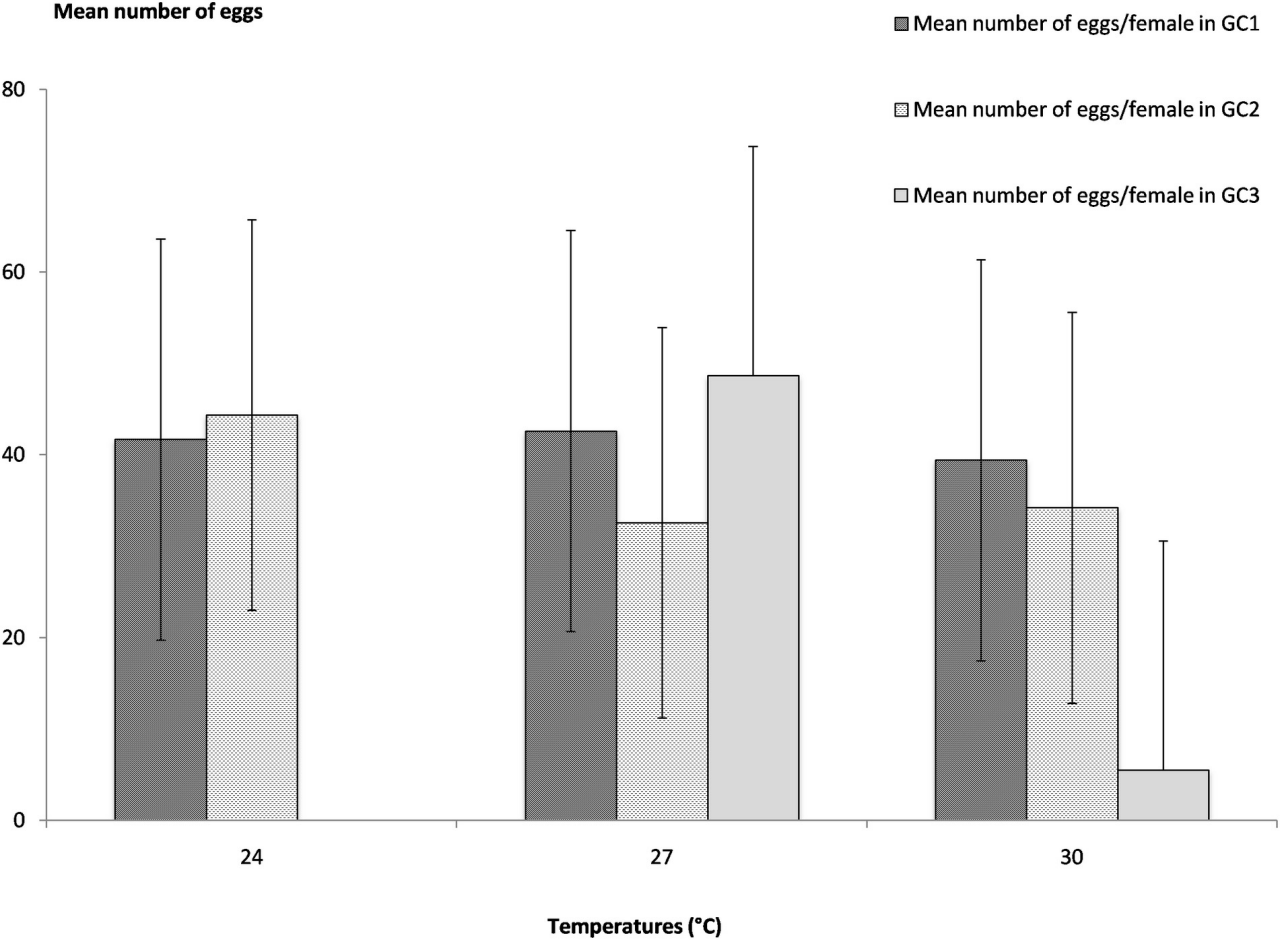
Mean numbers of eggs per female and per gonotrophic cycle at the different experimental temperatures of 24°C, 27°C and 30°C. GC1: first gonotrophic cycle, GC2: second gonotrophic cycle, GC3: third gonotrophic cycle.

The number of eggs deposited by each *Ae*. *aegypti* female varied greatly between each indiduals (S3 Table). The mean fecundity was 54 eggs/female, 62 eggs/per female and 53 eggs/female at 24°C, 27°C and 30°C respectively and no significant differences in the *Ae*. *aegypti* fecundity according to temperatures was estimated with ANOVA tests (S2 File, F_2,147_ = 0.57, *P ≤ 0*.*005*) and furthermore, the mean number of eggs/female and per gonotrophic cycle (Fig 5) again did not show significant differences between the first and the second GCs whatever the temperatures (S3 File) but a low mean number of eggs was observed for the third GC at 30°C. For the temperatures tested in the experiments, the fecundity of *Ae*. *aegypti* females does not vary with temperatures in a certain range, above 23°C. But, for the higher temperature, the fecundity varied according to the gonotrophic cycle number and consequently the age of female. The fecundity of *Ae*. *aegypti* females will impact on the population density and from the above results, the best performance is observe at 27°C, which supports that this is the optimal temperature for *Ae*. *aegypti* reproduction in Guadeloupe, in agreement with *Ae*. *aegypti* parity rates.

### Survival of females

**Fig 6:**
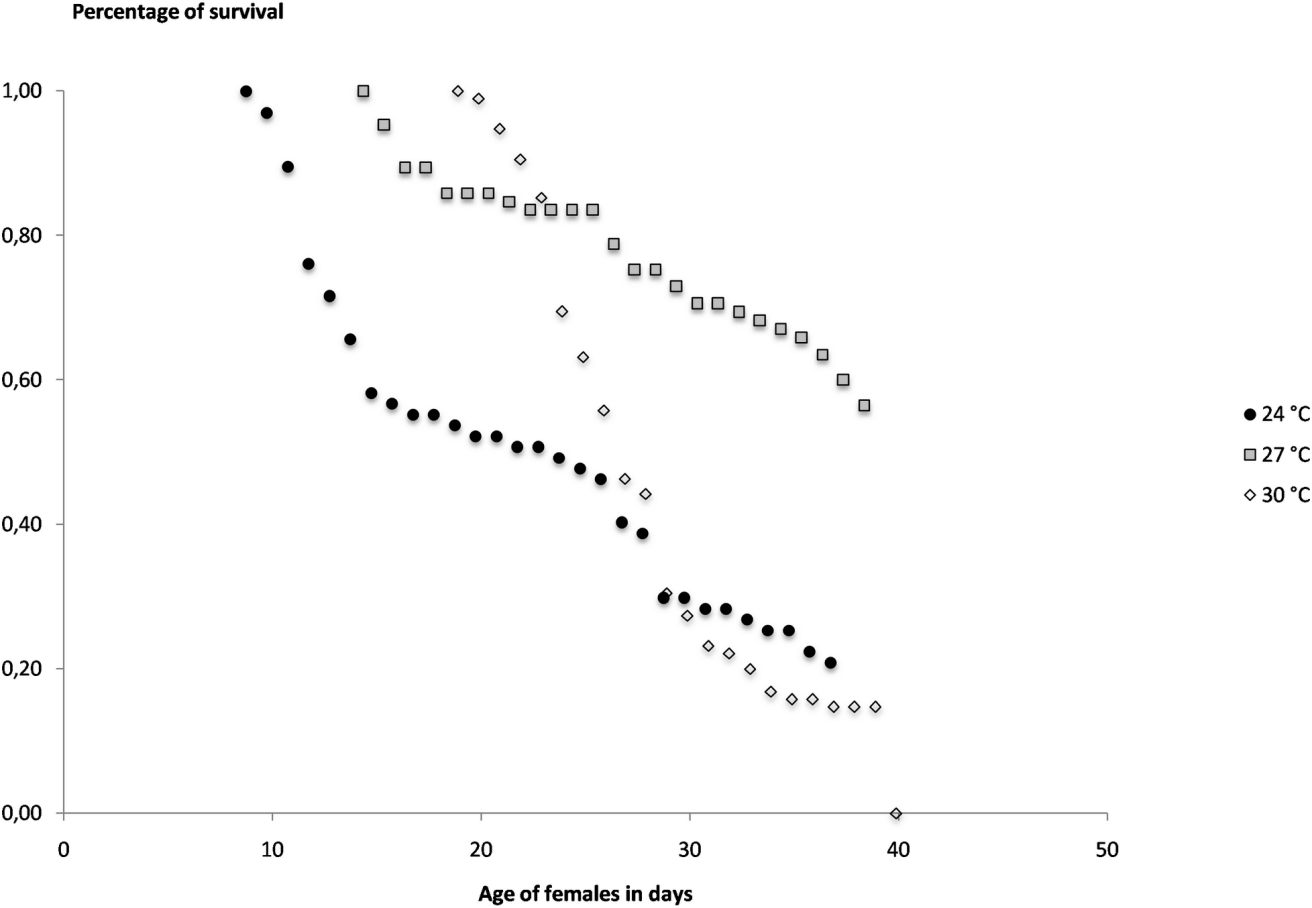
Survival of females of *Aedes aegypti* in laboratory conditions at constant temperatures of 24°C, 27°C and 30°C plotted against the age of females.

**Fig 7:**
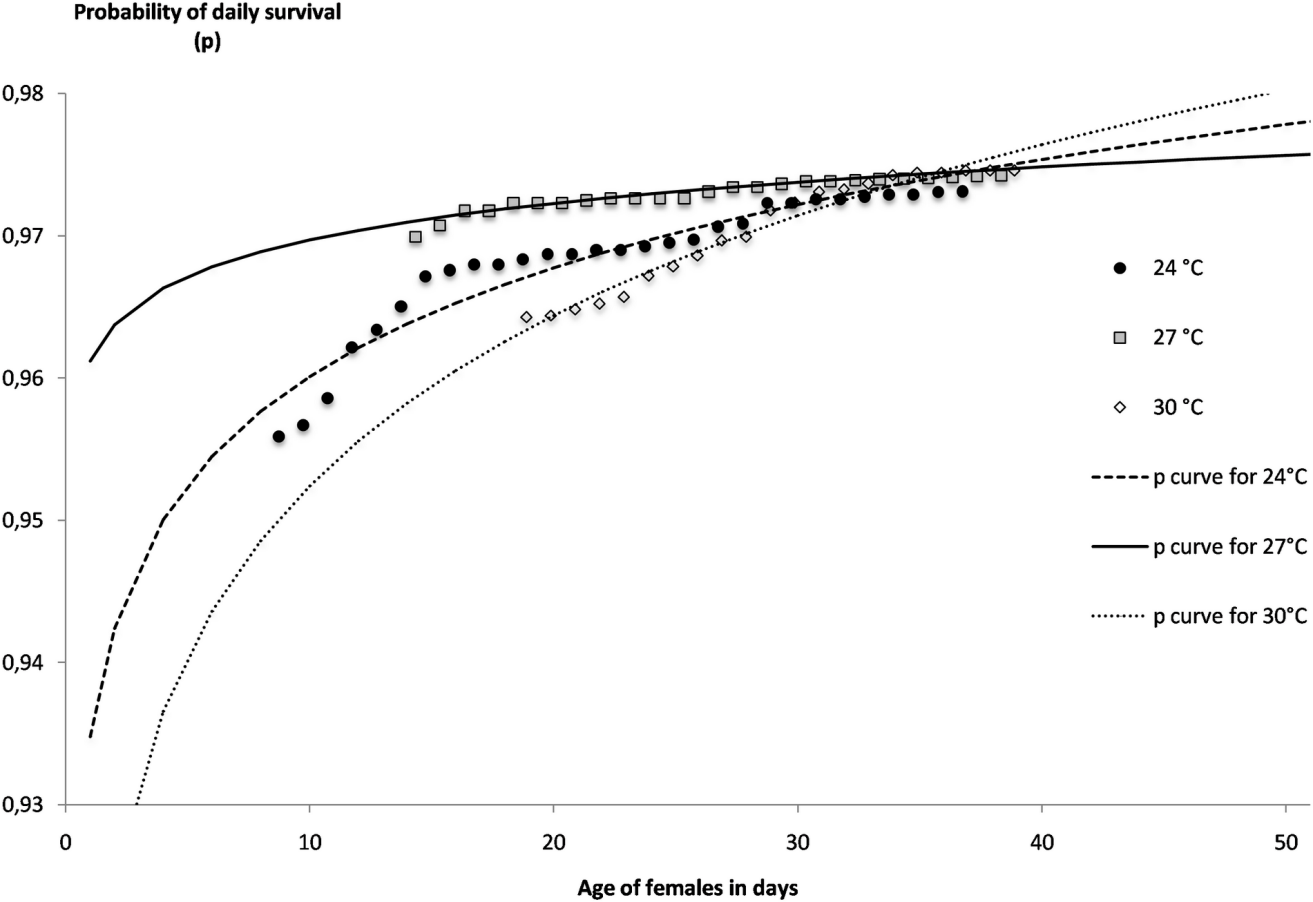
Probability of daily survival of females of *Aedes aegypti* in laboratory conditions at constant temperatures of 24°C, 27°C and 30°C plotted against the age.

The overall survival of the females through the different GCs and egg-laying decreases with time as expected, but differently according to the temperatures (Fig 6). At the lowest temperature (24°C) the female survival decreases early between day 10 and day 20 and half of the females do not survive more than 25 days. At the highest temperature (30°C), although the survival decreases after 20 days, the pattern is similar and half of the females do not survive more than 25 days with a very sharp mortality around days 20 to 25 (S4 Table). The best survival is obtained at the medium temperature of 27°C because 84% of females are still alive after 25 days and more than 50% are surviving more than 38 days. The survival rates of the females at the age of 25 days were compared by statistical analysis and were found significantly different between each of the experimental temperatures (Table 1). The daily probability of survival estimated from the mean life expectation plotted against the age of females shows that this probability is very different according to temperature for the youngest females (Fig 7). Again, the best daily survival is found at 27°C and the lowest survival is found at the highest temperature of 30°C. This result means that the younger females, in particular those aged less than 10 days old, will have a much shorter expectation of life at the extreme temperatures. For example a female of 10 days old will have an expectation of life of 24.5 days, 32.8 days and 20.3 days at 24°C, 27°C and 30°C respectively. For the lower temperature, this reduced life duration may be critical to achieve pathogens transmission. For each day (i) of the experiments and each temperature, the daily probability of survival of the *Ae*. *aegypti* females (s_i_) could be extrapolated from the p curves in Fig 7 which are logarithmic regression of survival according to the age of the females (a_i_), with:

Si=0.011ln(ai)+0.934,for24°C

Si=0.003ln(ai)+0.961,for27.0°C

Si=0.017ln(ai)+0.912,for30°C

**Fig 8:**
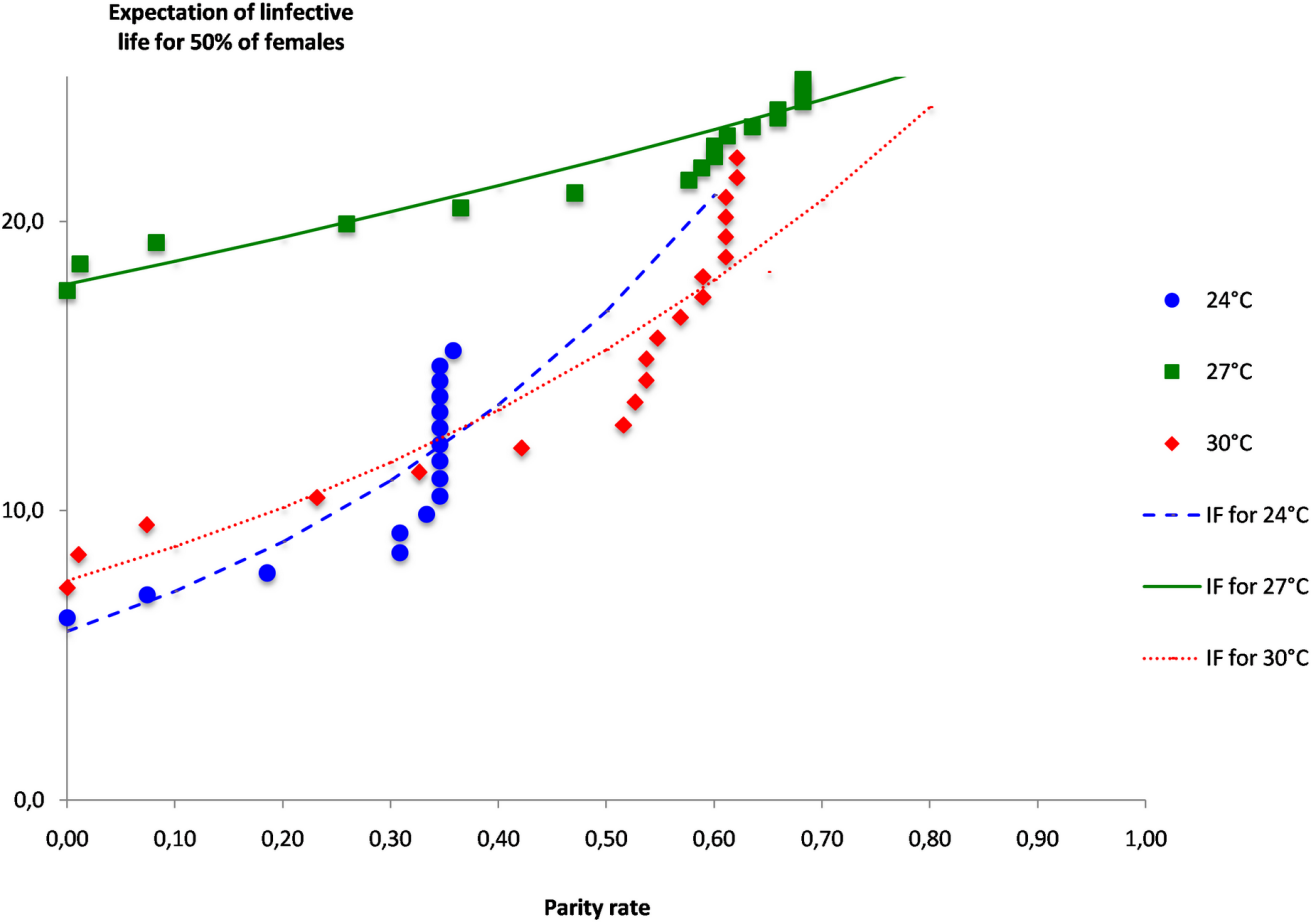
Expectation of infective life (I_i_) in days for 50% of the females of *Aedes aegypti* females according to the EIP at constant temperatures of 24°C, 27°C and 30°C, plotted against the parity rates.

When the daily probability of survival are transformed into expectation of life, the model represented in Fig 8 shows that at 24°C, the expectation of life is more than 20 days even for low parity rates and increases until about 30 days when parity rates are above 30%. For the medium temperature of 27°C, the expectation of life is maximum at 30 days, even for the lowest parity rates and increases sharply to 40 days when parity rates are about 60%. For the highest temperature of 30°C, the expectation of life is unexpectedly low when parity rates are low, with less than 20 days for parity rates of about 10% and increases slowly for parity rates less than 50%, but when parity rates are above this value the expectation of life increases sharply to reach about 30 days when parity rate reach 60%. The model representing the evolution of the expectation of life according to parity rates shows that the longest life expectation is obtained at 27°C

### Expectancy of infective life and transmission risk according to parity rates

As expected, the duration of infective life, which is the number of days the female mosquito can transmit the disease, increases when the parity rates are increasing (Fig 8), for all temperatures studied, indicating that high field parity rates are representing an increasing risk of transmission due to the potential female infective life. However, this potential infective life has different patterns according to temperature. At the lower temperature of 24°C, the duration of infective life remain less than 10 days for parity rates of less than 30%, and suddenly increases between 30% and 40% to reach a maximum of about 15 days. At the medium temperature of 27°C, the duration of infective life is very high with about 18 days even if the parity rates are very low, and increases slowly to reach a maximum duration of about 24 days when 70% of the females are parous. Finally, at the highest temperature of 30°C, the pattern is similar to what is observed at the low temperature with short duration of infective life when parity rates are lower, with about 10 days of potential infective life when 10% of the female are parous. But when parity rates are more than 50%, then the duration of potential infective life increases sharply to reach value of more than 20 days. In terms of transmission risks, the medium temperature of 27°C can maintain high transmission risks due to a long duration of potential infective life, even for low parity rates. Contrarily for low temperature, transmission risks remain also low for low parity rates and never reach the longest values, but for high temperature, high parity rates are correlated with high transmission risks again due to a long duration of potential infective life.

### Observation of ovaries during the egg maturation

**Fig 9:**
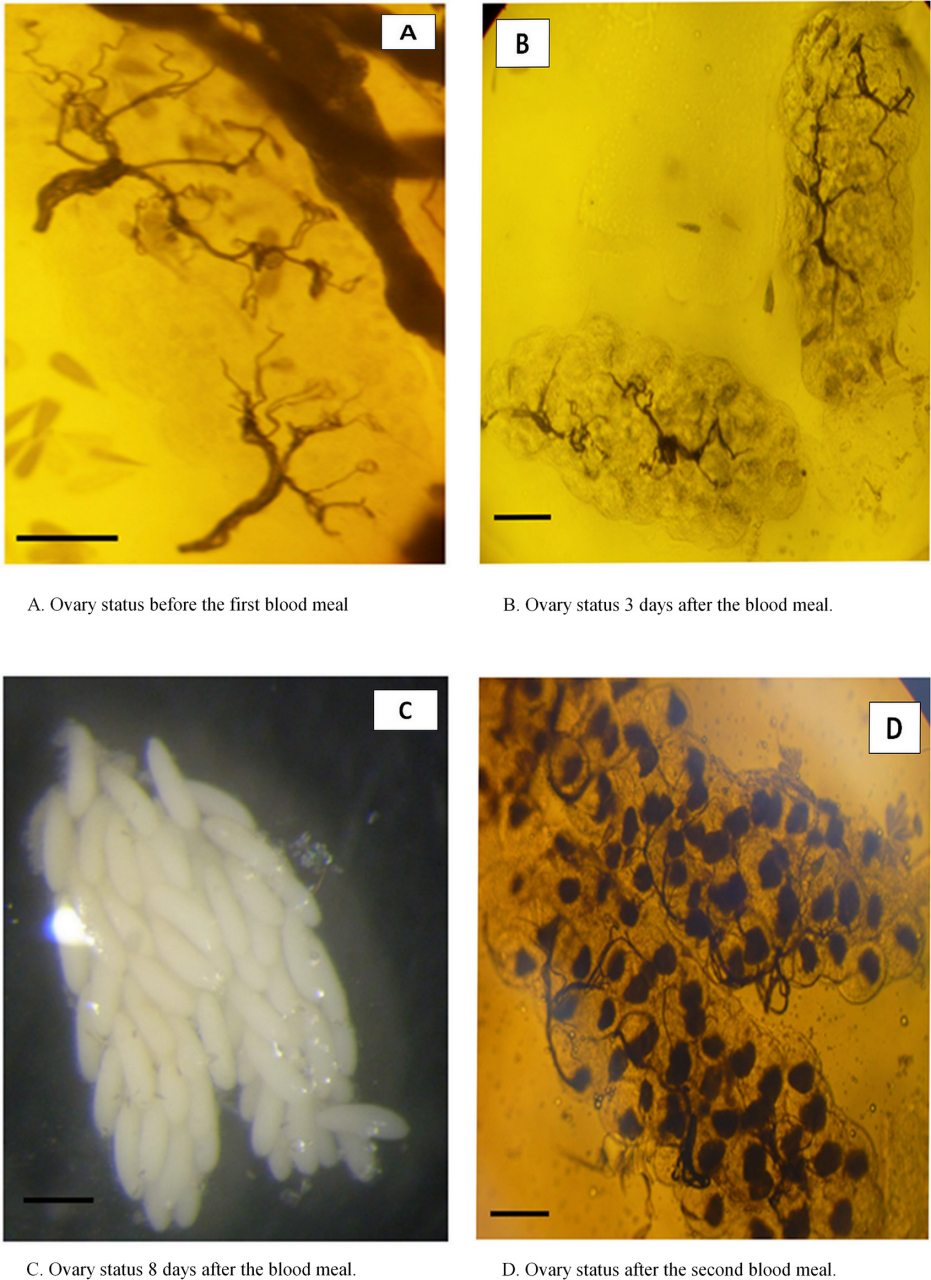
Histological pictures (x40) from binocular loupe observation of the ovaries of selected females of *Aedes aegypti* during the gonotrophic cycle at 24°C. Before the first blood meal when the female is nulliparous **(A)**, 3 days after the blood meal when eggs are forming **(B)**, about 8 days after the blood meal when the female is gravid and ready to lay the eggs **(C)** and after the second blood meal when the female is parous and the second batch of eggs in formation **(D)**. Scale bars, 0.25 mm.

When collected in the field, the females of *Ae*. *aegypti* can be classified into 4 groups according to their physiological status [34] from unfed with a flat abdomen, blood-fed with a red and distended abdomen, semi-gravid when the abdomen is half-black and gravid when the abdomen is distented by mature eggs, ready to be deposited. Before the first batch of eggs is laid, the females are called nulliparous and after the first batch of eggs is laid females are called parous [35]. The determination of the parity status from the ovaries observation of field collected *Ae*. *aegypti* mosquitoes is rather difficult and requires high standard material and training [36]. The experiments carried out to study the GC and fecundity give us the opportunity to observe the ovaries of females with a known status since the mosquito females were kept separately. When females are nulliparous and unfed, the ovaries are almost transparent and the tracheoles are grouped into small balls in the center of the ovaries (Fig 9A). The loops are clearly visible at the top of the tracheoles. After the first blood meal, the females are still nulliparous but the maturation of the eggs shades the ovaries and hide in some portion the tracheoles loops (Fig 9B). The nulliparous status is thus less evident. However, when some loops can be seen, the nulliparous status can be assigned. At the end of the egg maturation, even when the females are nulliparous, the ovaries are full of mature eggs and the tracheoles cannot be seen (Fig 9C), the parity status remains then undetermined. At the beginning of the second gonotrophic cycle, the females are parous and when the ovaries start maturing the following batch of eggs, the tracheoles can be observed again and no more loops are visible (Fig 9D). In the contrary, the tracheoles are well extented and are seen in ranging in all the ovaries. This type of extented tracheoles allow the determination of the parous status. When females of *Ae*. *aegypti* are collected in the field the dissection of ovaries will result in the determined nulliparous and parous females and undetermined females. The parity status will then be related to the female survival and expectation of infective life for a better appreciation of the transmission risks.

## Discussion

The main findings on the parity rates increase at different experimental constant temperatures indicate that our knowledge of the blood-taking behavior of *Ae*. *aegypti* females needs to be revised. It is usually accepted that almost all females will take a blood-meal to complete the gonotrophic cycles [37], but our results show that under laboratory conditions blood-taking is never 100% and is strongly temperature dependent. This observation was previously reported for the second blood-meal [38] but this is the first time that this result is observed for the first blood-meal. It is also usually accepted that most of the female that will complete a blood meal will lay eggs, however our results show that true parity rates on the engorged females again never reach 100%. Consequently, true *Ae*. *aegypti* parity rates are very different from the ones found in some field collections [39, 40]. This difference may be due to the type of trap, the period of collection and also the difficulty to accurately estimate the parity from ovary dissection [36]. On another hand, the parity rates found in our experiments are probably not optimum due to constant temperatures, since fluctuating temperatures have been shown to be more favorable [41]. And further the containment of the females in single containers may not be favorable to a normal behavior. Nevertheless temperatures influence the fluctuation of the *Ae*. *aegypti* parity rates as for other *Ae*. *aegypti* biological traits [42, 43] and this observation has to be taken into account when using field parity rates to estimate transmission risks.

The duration of the gonotrophic cycles is consistent with previous observations also shorter durations have been described [44]. Under natural conditions the multiple blood-feeding of *Ae*. *aegypti* mosquito within each gonotrophic cycle [45] results in some difficulty to recognize the number of gonotrophic cycles. In addition blood-feeding and egg-laying may occur in a non-sequential pattern [46]. This behavior has an important epidemiological consequence since the transmission can occur at any time of the mosquito life after the extrinsic incubation period has been completed. Further, the above results show that not all females will take another blood-meal after egg-laying with a very small percentage at low temperature and a percentage of about 40% at suitable temperatures (S5 Table). This may indicate that aged females will tend to decrease their biting habits. Consequently the higher infectivity of aged females is balanced by their feeding behavior, which may explain why high parity rates are not correlated with high transmission (personal unpublished data). Surprisingly, the fecundity of *Ae*. *aegypti* was not affected by constant temperature since the mean numbers of eggs laid by females did not vary significantly according to temperature. However, the influence of temperature on fecundity may be more relevant with fluctuating temperatures [47].

The main objective of the above experiments were to model the expectation of infective life of the females of *Ae*. *aegypti* from the parity rates The current surveillance of the dengue vector mosquitoes and thus the transmission risks uses several larval and pupae indices, but these methods are not very efficient in the prevention of dengue epidemics, maybe because immature mosquito stages are not the transmitting ones. Thus, a surveillance of the adult and in particular the aged females could be better related to transmission risks. The model could then be used to estimate the virus transmission risks from field parity rates, as this is the case for the malaria mosquito *Anopheles gambiae* and the malaria transmission risks [24]. The female infective life starts when the extrinsic incubation period, also temperature dependent [48], is completed. When compared to the *An*. *gambiae* model, the estimated expectations of life and of infective life of *Ae*. *aegypti* are strongly different (Table 1). For the same temperature and the same parity rates the *Ae*. *aegypti* expectation of infective life is ten times longer, and thus transmission risks are much more important. This result enhances the fact that specific studies on *Ae*. *aegypti* expectation of life are necessary before using field parity rates to estimate the entomological risks of a dengue epidemic. Data obtained with other mosquito species and maybe from the same species but originated from another geographical location may lead to an important underestimation of the life duration, as exemplified in Table 3. The difference of expectations of infective life between *Ae*. *aegypti* and *An*. *gambiae* at the same temperature and for the same parity rates can be included in the factors that explain why the dengue transmission cycle is so active and thus difficult to interrupt [49].

**Table 3:** Expectations of life (in days) and expectations of infective life (in days) for *An*. *gambiae* and for *Ae*. *aegypti* from the parity rates.

Study	Coz (1961)	Garrett-Jones (1964)	Present Study	
Mosquito species	*An*. *gambiae*	*An*. *gambiae*	*Ae*. *aegypti*	*Ae*. *aegypti*
Temperature (°C)	25	30	27	30.7
Parity rate	0.65	0.65	0.65	0.65
Expectation of life (days)	9.5	9.49	34.35	26
**Expectation of infective life (days)** ^a^	**2 to 4**	**2 to 4**	**23.24**	**18.26**

*An*. *gambiae* models are based on Coz & al. (1961) and Garret-Jones and Grab (1964) publications.

^a^For *Ae*. *aegypti* and dengue virus EIP is estimated from Carrington et al. (2013)

On another hand, the experiments described herein were a good opportunity to directly observe the *Ae*. *aegypti* female ovary at different times from the female birth to the blood-feeding and during the egg maturation. Nulliparous females are quite easy to identify since the enrolled tracheoles are visible in the dissection [50], this is also the case just after the blood-feeding. However, when the egg maturation has begun it is almost impossible to distinguish the parity as well as the developmental stage of the eggs. Again, just after egg laying, the parous females are also easily identified since the tracheoles are developed and well visible. Consequently, when collecting females in the field, a great number of them cannot be associated to a parity status. This is a further complication to estimate the parity rates and thus the female expectation of life. A more reasonable approach would be to consider females that are strictly nulliparous, even if in the parous group some of them would be in the process of developing their first batch of eggs [36]. Then the parity rate may be overestimated as well as the expectation of infective life.

Finally, the last missing knowledge in the estimation of the transmission risks from an entomological indicator is how the *Ae*. *aegypti* expectation of life is related to the real transmission of the viruses among the human population. For this purpose, longitudinal studies of field parity rates transformed into expectation of infective life according to EIP and temperatures should be carried out before, during and after an epidemic episode.

## Supporting Information

S1 File ANOVA for testing the difference between the durations of the first, second and third gonotrophic cycles at each experimental temperature. The duration of GC1 and GC2 were significantly different according to temperature, for GC1, F_2,147_ = 34.60, *P ≤ 0*.*005*; for GC2, F_2,56_ = 6.52, *P ≤ 0*.*005*.
(PDF)

S2 File ANOVA for testing the difference between the number of eggs at each experimental temperature. No significant differences in the *Ae*. *aegypti* fecundity according to temperatures was estimated with ANOVA tests (F_2,147_ = 0.57, *P ≤ 0*.*005*).
(PDF)

S3 File Kruskall-Wallis Analysis for testing the difference between the mean numbers of eggs produced at gonotrophic cycles one and two. No significant differences in the *Ae*. *aegypti* fecundity according to gonotrophic cycle was found.
(PDF)

S1 Table Numbers of parous *Aedes aegypti* females after the first blood meal and according to time, for the 3 temperatures 24°C, 27°C and 30°C. The numbers of parous females allow the calculation of the mean durations of gonotrophic cycles and the percentage of parity according to time at each temperature. These values are used in Figs 2A and2B.
(PDF)

S2 Table Durations of each gonotrophic cycle for each *Aedes aegypti* females after the all blood meals for the 3 temperatures 24°C, 27°C and 30°C. These data allow the calculation of the mean number of gonotrophic cycle, with their standard deviation, the mean duration of the all gonotrophic cycles, as well as the mean duration of the first, second and third gonotrophic cycle with their respective standard deviation, at each temperature. These values are used in Figs 3 and 4.
(PDF)

S3 Table Numbers of eggs laid by each females at the first, second and third gonotrophic cycles and at each experimental temperature. These data allow the calculation of the mean number of eggs per female in their life, and for each gonotrophic cycle, with their standard deviation, at each temperature. These values are used in Fig 5.
(PDF)

S4 Table Survival and age of *Aedes aegypti* females for each day of the experiments at each experimental temperature. These data allow the calculation of survival rates, mortality rates, duration of life and daily probability of survival. These data are used in the modeling of daily probability of survival according to the age of the females at each experimental temperatures. These values are used in Figs 6 and 7.
(PDF)

S5 Table Overall percentages of females taking a second blood-meal according to temperatures and consequences on pathogens transmission risks. The percentage of female potentially able to transmit the virus increases from 28% at 24°C to 43% and 39% at 27°C and 30°C respectively.
(PDF)

## References

[1] Gubler DJ (2002) Epidemic dengue/dengue hemorrhagic fever as a public health, social and economic problem in the 21st century. Trends Microbiol 10: 100–103. 11827812 10.1016/s0966-842x(01)02288-0

[2] Laughlin CA, Morens DM, Cassetti MC, Costero-Saint Denis A, San Martin JL, Whitehead SS et al (2012) Dengue research opportunities in the Americas. J Infect Dis 206: 1121–1127. 10.1093/infdis/jis351 22782946 PMC3499110

[3] Strobel M, Jattiot F, Boulard F, Lamaury I, Salin J, Jarrige B et al (1998) Emergence of dengue hemorrhagic fever in French Antilles. 3 initial fatal cases in Guadeloupe. Presse Med 27: 1376–1378. 9793052

[4] Gharbi M, Quenel P, Gustave J, Cassadou S, La Ruche G, Girdary L et al (2011) Time series analysis of dengue incidence in Guadeloupe, French West Indies: forecasting models using climate variables as predictors. BMC Infect Dis 11:166 10.1186/1471-2334-11-166 21658238 PMC3128053

[5] Larrieu S, Cassadou S, Rosine J, Chappert JL, Blateau A, Ledrans M et al (2013) Lessons raised by the major 2010 dengue epidemics in the French West Indies. Acta Trop 131: 37–40. 10.1016/j.actatropica.2013.11.023 24315801

[6] Gustave J, Fouque F, Cassadou S, Leon L, Anicet G, Ramdini C et al (2012) Increasing Role of Roof Gutters as *Aedes aegypti* (Diptera: Culicidae) Breeding Sites in Guadeloupe (French West Indies) and Consequences on Dengue Transmission and Vector Control. J Trop Med 2012:249524 10.1155/2012/249524 22548085 PMC3323855

[7] Cassadou S, Boucau S, Petit-Sinturel M, Huc P, Leparc-Goffart I, Ledrans M (2014) Emergence of chikungunya fever on the French side of Saint Martin island, October to December 2013. Euro Surveill 19(13) pii: 20752. 10.2807/1560-7917.es2014.19.13.20752 24721536

[8] CIRE Antilles-Guyane (2014) Le chikungunya dans les Antilles-Guyane. Le point épidémiologique N°34. ARS/INVS.

[9] Higgs S (2014) Chikungunya virus: a major emerging threat. Vector-Borne and Zoonotic Diseases, 14 (8): 1–2. 25029622 10.1089/vbz.2014.14.8.edit

[10] David MR, Ribeiro GS, Freitas RM (2012) Bionomics of *Culex quinquefasciatus* within urban areas of Rio de Janeiro, Southeastern Brazil. Rev Saude Publica 46: 858–865. 23128263 10.1590/s0034-89102012000500013

[11] Davidson G (1954) Estimation of the survival-rate of anopheline mosquitoes in nature. Nature 174: 792–793. 13214009 10.1038/174792a0

[12] Lehane MJ (2005) The biology of blood-sucking insects Cambridge: University Press. 321p.

[13] Scott TW, Takken W (2012) Feeding strategies of anthropophilic mosquitoes result in increased risk of pathogen transmission.Trends Parasitol 28:114–121. 10.1016/j.pt.2012.01.001 22300806

[14] Kuno G (1995) Review of the factors modulating dengue transmission. Epidemiol Rev 17:321–335. 8654514 10.1093/oxfordjournals.epirev.a036196

[15] Klowden MJ (1995) Blood, sex and mosquitoes. The mechanism that control mosquito blood feeding behavior. Bioscience 45: 326–331.

[16] Clements AN (1992) The Biology of Mosquitoes: Development, nutrition, and reproduction, Chapman & Hall. 509p.

[17] Scott TW, Clark GG, Lorenz LH, Amerasinghe PH, Reiter P, Edman JD (1993) Detection of multiple blood feeding in *Aedes aegypti* (Diptera: Culicidae) during a single gonotrophic cycle using a histologic technique. J Med Entomol 30: 94–99. 8433350 10.1093/jmedent/30.1.94

[18] Pant CP, Yasuno M (1973) Field studies on the gonotrophic cycle of *Aedes aegypti* in Bangkok, Thailand. J Med Entomol 10: 219–23. 4707760 10.1093/jmedent/10.2.219

[19] Saifur RGM, Dieng H, Hassan AA, Salmah MRC, Satho T, Miake F et al (2012) Changing Domesticity of *Aedes aegypti* in Northern Peninsular Malaysia: Reproductive Consequences and Potential Epidemiological Implications. PLoS ONE 7(2): e30919 10.1371/journal.pone.0030919 22363516 PMC3281890

[20] Lambrechts L, Paaijmans KP, Fansiri T, Carrington LB, Kramer LD, Thomas MB et al (2011) Impact of daily temperature fluctuations on dengue virus transmission by *Aedes aegypti* . Proc Natl Acad Sci U S A 108: 7460–7465. 10.1073/pnas.1101377108 21502510 PMC3088608

[21] Carrington LB, Armijos MV, Lambrechts L, Scott TW (2013) Fluctuations at a low mean temperature accelerate dengue virus transmission by *Aedes aegypti* . PLoS Negl Trop 7(4): e2190. 10.1371/journal.pntd.0002190 PMC3636080 23638208

[22] Scott TW, Chow E, Strickman D, Kittayapong P, Wirtz RA, Lorenz LH et al (1993) Blood-feeding patterns of *Aedes aegypti* (Diptera: Culicidae) collected in a rural Thai village. J Med Entomol 30: 922–927. 8254642 10.1093/jmedent/30.5.922

[23] MacDonald G (1952) The measurement of malaria transmission. Proc R Soc Med. 48: 295–302. 10.1177/003591575504800409 PMC1918775 14371594

[24] Garrett-Jones C, Grab B (1964) The assessment of insecticidal impact on the malaria mosquito's vectorial capacity, from data on the proportion of parous females. Bull World Health Organ 31: 71–86. 14230896 PMC2555159

[25] Fouque F, Carinci R, Gaborit P, Issaly J, Bicout DJ, Sabatier P (2006) *Aedes Aegypti* survival and Dengue transmission patterns in French Guiana. Journal of Vector Ecology 31: 390–399. 17249358 10.3376/1081-1710(2006)31[390:aasadt]2.0.co;2

[26] Maciel-de-Freitas R, Codeço CT, Lourenço-de-Oliveira R (2007). Daily survival rates and dispersal of *Aedes aegypti* females in Rio de Janeiro, Brazil. Am J Trop Med Hyg 76: 659–665. 17426166

[27] Harrington LC, Françoisevermeylen, Jones JJ, Kitthawee S, Sithiprasasna R, Edman JD et al (2008) Age-dependent survival of the dengue vector *Aedes aegypti* (Diptera: Culicidae) demonstrated by simultaneous release-recapture of different age cohorts. J Med Entomol 45: 307–313. 18402147 10.1603/0022-2585(2008)45[307:asotdv]2.0.co;2

[28] Morrison AC, Costero A, Edman JD, Clark GG, Scott TW (1999) Increased fecundity of *Aedes aegypti* fed human blood before release in a mark-recapture study in Puerto Rico. J Am Mosq Control Assoc. 15:98–104. 10412105

[29] Detinova TS (1963) Méthodes à appliquer pour classer par groupe d'âge les diptères présentant une importance médicale: Notamment certains vecteurs du paludisme. WHO Suisse. pp. 49–52.

[30] Zar JH (1984) Biostatistical Analysis. 2nd edn. New Jersey: Prentice Hall.

[31] MacDonald G (1956) Epidemiological basis of malaria control. Bull Wld Hlth Org 15: 613–626. PMC2538278 13404439

[32] Reisen WK, Fang Y, Martinez VM (2006) Effects of temperature on the transmission of West Nile Virus by *Culex tarsalis* (Diptera: Culicidae). J Med Entomol 43:309–317. 16619616 10.1603/0022-2585(2006)043[0309:EOTOTT]2.0.CO;2

[33] Harrington LC, Fleisher A, Ruiz-Moreno D, Vermeylen F, Wa CV, Poulson RL et al Heterogeneous feeding patterns of the dengue vector, *Aedes aegypti*, on individual human hosts in rural Thailand. PLoS Negl Trop Dis. 2014 8 7;8(8):e3048 10.1371/journal.pntd.0003048 25102306 PMC4125296

[34] Coz J, Gruchet H, Chauvet G, Coz M (1961) Estimation of survival rates in Anopheles species. Bull Soc Pathol Exot Filiales 54: 1353–1358. 13881923

[35] Clements AN (1992) The Biology of Mosquitoes: Development, Nutrition and Reproduction. CABI Publishing, Chapman, & Hall.

[36] Joy TK, Jeffrey Gutierrez EH, Ernst K, Walker KR, Carriere Y, Torabi M et al (2012) Aging field collected *Aedes aegypti* to determine their capacity for dengue transmission in the southwestern United States. PLoS One 7(10):e46946 10.1371/journal.pone.0046946 23077536 PMC3470585

[37] Christophers RS (1960) *Aedes aegypti* (L.) the yellow fever mosquito: its life history, bionomics and structure Cambridge University Press, 739 pp.

[38] Chadee DD (2012) Studies on the post-oviposition blood-feeding behaviour of *Aedes aegypti* (L.) (Diptera: Culicidae) in the laboratory. Pathog Glob Health 106(7): 413–417. 10.1179/2047773212Y.0000000036 23265613 PMC4001624

[39] Chadee DD, Ritchie SA (2010) Oviposition behaviour and parity rates of *Aedes aegypti* collected in sticky traps in Trinidad, West Indies. Acta Trop. 116(3): 212–216. 10.1016/j.actatropica.2010.08.008 20727339

[40] Lima-Camara TN, Honório NA, Lourenço-de-Oliveira R (2007) Parity and ovarian development of *Aedes aegypti* and *Ae*. *albopictus* (Diptera: Culicidae) in metropolitan Rio de Janeiro. J Vector Ecol. 32(1): 34–40. 17633424 10.3376/1081-1710(2007)32[34:paodoa]2.0.co;2

[41] Carrington LB, Armijos MV, Lambrechts L, Barker CM, Scott TW (2013) Effects of fluctuating daily temperatures at critical thermal extremes on *Aedes aegypti* life-history traits. PLoS One 8(3):e58824 10.1371/journal.pone.0058824 23520534 PMC3592833

[42] Mohammed A, Chadee DD (2011) Effects of different temperature regimens on the development of *Aedes aegypti* (L.) (Diptera: Culicidae) mosquitoes. Acta Trop. 119(1): 38–43. 10.1016/j.actatropica.2011.04.004 21549680

[43] Yang HM, Macoris ML, Galvani KC, Andrighetti MT, Wanderley DM (2009) Assessing the effects of temperature on the population of *Aedes aegypti*, the vector of dengue. Epidemiol Infect. 137(8): 1188–1202. 10.1017/S0950268809002040 19192322

[44] Wong J, Astete H, Morrison AC, Scott TW (2011) Sampling considerations for designing Aedes aegypti (Diptera:Culicidae) oviposition studies in Iquitos, Peru: substrate preference, diurnal periodicity, and gonotrophic cycle length. J Med Entomol. 48(1): 45–52. 21337947 10.1603/me10149 PMC3108245

[45] Scott TW1, Amerasinghe PH, Morrison AC, Lorenz LH, Clark GG, Strickman D et al (2000) Longitudinal studies of *Aedes aegypti* (Diptera: Culicidae) in Thailand and Puerto Rico: blood feeding frequency. J Med Entomol. 37(1): 89–101. 15218911 10.1603/0022-2585-37.1.89

[46] Farjana T, Tuno N (2013) Multiple blood feeding and host-seeking behavior in *Aedes aegypti* and *Aedes albopictus* (Diptera: Culicidae). J Med Entomol. 50(4): 838–846. 23926783 10.1603/me12146

[47] Carrington LB, Seifert SN, Willits NH, Lambrechts L, Scott TW (2013) Large diurnal temperature fluctuations negatively influence *Aedes aegypti* (Diptera: Culicidae) life-history traits. J Med Entomol. 50(1): 43–51. 23427651 10.1603/me11242

[48] Watts DM, Burke DS, Harrison BA, Whitmire RE, Nisalak A (1987) Effect of Temperature on the Vector Efficiency of *Aedes aegypti* for Dengue 2 Virus. Am J Trop Med Hyg 36:143–152 3812879 10.4269/ajtmh.1987.36.143

[49] Reiner RC, Stoddard ST, Forshey BM, King AA, Ellis AM, Lloyd AL et al (2014) Time-varying, serotype-specific force of infection of dengue virus. Proc Natl Acad Sci U S A. 111(26): E2694–702. 10.1073/pnas.1314933111 24847073 PMC4084484

[50] Hugo LE, Quick-miles S, Kay BH, Ryan PA (2008) Evaluations of Mosquito Age Grading Techniques Based on Morphological Changes. J Med Entomol. 45(3): 353–369. 18533427 10.1603/0022-2585(2008)45[353:eomagt]2.0.co;2

