# Supplementary material for: Parity and Longevity of Aedes aegypti According to Temperatures in Controlled Conditions and Consequences on Dengue Transmission Risks
Source: PLoS One. 2015 Aug 10;10(8):e0135489. doi: 10.1371/journal.pone.0135489 (PMC4530937; doi:10.1371/journal.pone.0135489)
Supplement: S1 File — The duration of GC1 and GC2 were significantly different according to temperature, for GC1, F2,147 = 34.60, P ≤ 0.005; for GC2, F2,56 = 6.52, P ≤ 0.005. (PDF) [file pone.0135489.s001.pdf]

ANOVA one way statistical test

H0 : Temperature has no influence on duration of gonotrophic cycle

Mean duration of gonotrophic cycle 1

	24°C	27°C	30°C
1	9,67	8,5	4
2	9	9,67	7
3	13,67	5,5	5
4	8,5	8,75	5,5
5	9	8	4,5
6	10,5	13	5
7	9	7,5	8,33
8	9	11	5
9	15,33	16	7
10	10	16	6
11	9	9,25	6,5
12	8	8	6
13	10,33	5	7,33
14	10,33	9,33	4
15	8	11	4
16	14	9,71	4
17	13	22	5
18	10,33	10,67	6
19	7	13,33	5
20	13,33	20	6
21	8,5	15	5
22	8	14,5	6
23	7	11	7
24	13,4	5	4
25	21	5	10
26	11	8,67	5
27	8	5	5,5
28	8,5	4	5
29	13	5	4
30		11,25	6
31		7,75	5
32		3	5
33		7	5
34		8	6
35		12	6
36		6	4,5
37		10	5
38		11	5,5
39		5	5
40		9	7
41		5	6,5
42		6,5	6,5
43		7	6
44		7	4
45		6	5,5
46		15,57	7
47		6	5,5
48		6	5,5
49		11,25	5
50		5	5
51		13	5
52		8	5
53		16	7
54		6	5
55		7	6
56		4	6
57		4,5	5
58		8	5
59		9,25	5
60		9,5	5
61			8

Ti	305,39	545,95	341,16	1192,5
ni	29	60	61	150
xi barre	10,5307	9,09917	5,59279	25,2226
Ti carré	93263,1	298061	116390	507715
Ti carré/r	3215,97	4967,69	1908,04	10091,7

SCI = 611,318

SCT = 1909,85

SCE = SCT-SCI
SCE = 1298,54

Vc = SCI/k-1
Vc = 305,659

Ve = SCE/n-k
Ve = 8,83358

F = Vc/Ve
F = 34,6019

F 0,05 (2; 3,06

F > F alpha: we can't accept H0

Mean duration of gonotrophic cycle 2

24°C	27°C	30°C
7	9	4
8	4,5	4
9	3	4
8	5	3,5
5	10	4
5	7	4
7,33	5	6,5
11,5	3	6,67
	5	4
	11,5	4
	8,5	9,67
	10,5	6
	8,67	4
	2	4
	4,5	7
	6	6
	8,5	4
	13,6	6,75
	8	4
	6	4
	9,67	4
	9	7,33
	8	4
	6	7
	6,5	
	6	
	9	

Ti	60,83	193,44	122,42	376,69
ni	8	27	24	59
xi barre	7,60375	7,16444	5,10083	19,869
Ti carré	3700,29	37419	14986,7	56106
Ti carré/r	462,536	1385,89	624,444	2472,87

SCI = 67,8642

SCT = 359,15

SCE = SCT-SCI
SCE = 291,286

Vc = SCI/k-1
Vc = 33,9321

Ve = SCE/n-k
Ve = 5,20153

F = Vc/Ve
F = 6,52349

3,15 < F alpha < 3,18 d'où F > F alpha: We can't accept H0
