# Supplementary material for: Parity and Longevity of Aedes aegypti According to Temperatures in Controlled Conditions and Consequences on Dengue Transmission Risks
Source: PLoS One. 2015 Aug 10;10(8):e0135489. doi: 10.1371/journal.pone.0135489 (PMC4530937; doi:10.1371/journal.pone.0135489)
Supplement: S2 File — No significant differences in the Ae. aegypti fecundity according to temperatures was estimated with ANOVA tests (F2,147 = 0.57, P ≤ 0.005). (PDF) [file pone.0135489.s002.pdf]

ANOVA one way statistical test

H0 : Temperature has no influence on the number of eggs

Mean number of eggs per female

	24°C	27°C	30°C
1	34	58	18
2	36,5	40	8
3	53	33,67	57
4	57,5	56	51,5
5	40	19	38
6	67	1	30
7	38	18,5	32
8	72	14	39,5
9	58	29	14
10	48	6	12
11	67,5	51	30,5
12	28	100	42
13	47,5	51	44
14	52	50	58
15	21	46	33
16	77	58	57
17	53	70	37
18	22	23,5	56
19	21	53	29
20	51	2	23
21	16	56	22,5
22	14	82	43
23	24	66,5	43
24	29	50,5	39
25	1	39	20
26	31	51	15
27	39	70	29,5
28	41	31,5	33
29	35	1	60
30		41	15
31		34	13
32		1	26
33		36	48
34		45	5
35		8	51
36		56	52,5
37		1	46
38		43	33
39		49	57
40		59	93
41		52,33	50,5
42		22	27
43		57	64,5
44		73	59,5
45		51	58
46		40	32
47		59,5	33,3
48		31	42
49		25	41,5
50		45,67	24
51		36	44
52		2	45
53		2	16
54		24	33
55		54,67	38,5
56		48,67	14
57		32,5	40
58		44	60
59		49	24
60		28	2
61			21

Ti	1174	2377,51	2223,8	5775,31
ni	29	60	61	150
xi barre	40,4827586	39,6251667	36,4557377	116,563663
Ti carré	1378276	5652553,8	4945286,44	11976116,2
Ti carré/ni	47526,7586	94209,23	81070,2695	222806,258

SCI = 444,88749

SCT = 57193,4439

SCE = 56748,5564

Vc = 222,443745

Ve = 386,044601

F = 0,57621255

F < F alpha (3,00): we can't reject H0
