# Supplementary material for: Parity and Longevity of Aedes aegypti According to Temperatures in Controlled Conditions and Consequences on Dengue Transmission Risks
Source: PLoS One. 2015 Aug 10;10(8):e0135489. doi: 10.1371/journal.pone.0135489 (PMC4530937; doi:10.1371/journal.pone.0135489)
Supplement: S3 File — No significant differences in the Ae. aegypti fecundity according to gonotrophic cycle was found. (PDF) [file pone.0135489.s003.pdf]

GC1	GC2
41,69	44,38
42,62	32,59
39,43	34,21

XLSTAT 2014.6.01 - Comparaison de k échantillons (Kruskal-Wallis, Friedman, ...) - le 07/07/2015 à 14:54:52
Echantillons : Classeur = Supplementary-Files-S1toS8-PONE-01616vuDG.xlsx / Feuille = Table S6 / Plage = 'Table S6'!$B$1:$C$4 / 3 lignes et 2 colonnes
Niveau de signification (%) : 5
p-value : p-value asymptotique

Statistiques descriptives :

Variable	Observations	Obs. avec données manquantes	Obs. sans données manquantes	Minimum	Maximum	Moyenne	Ecart-type
GC1	3	0	3	39,430	42,620	41,247	1,641
GC2	3	0	3	32,590	44,380	37,060	6,391

Test de Kruskal-Wallis :

K (Valeur observée)	0,429
K (Valeur critique)	3,841
DDL	1
p-value (bilatérale)	0,513
alpha	0,05

Une approximation a été utilisée pour calculer la p-value.

Interprétation du test :
H0 : Les échantillons proviennent de la même population.
Ha : Les échantillons proviennent de populations différentes.

Etant donné que la p-value calculée est supérieure au niveau de signification seuil alpha=0,05, on ne peut pas rejeter l'hypothèse nulle H0.

Le risque de rejeter l'hypothèse nulle H0 alors qu'elle est vraie est de 51,27%.

Comparaisons multiples par paires suivant la procédure de Steel-Dwass-Critchlow-Fligner / Test bilatéral :

Echantillon	Effectif	Somme des rangs	Moyenne des rangs	Groupes
GC2	3	9,000	3,000	A
GC1	3	12,000	4,000	A

Wij :

	GC1	GC2
GC1		0,926
GC2	-0,926	

Différences significatives :

	GC1	GC2
GC1		Non
GC2	Non	

p-values :

	GC1	GC2
GC1	**1**	0,513
GC2	0,513	**1**
