# Supplementary material for: Parity and Longevity of Aedes aegypti According to Temperatures in Controlled Conditions and Consequences on Dengue Transmission Risks
Source: PLoS One. 2015 Aug 10;10(8):e0135489. doi: 10.1371/journal.pone.0135489 (PMC4530937; doi:10.1371/journal.pone.0135489)
Supplement: S1 Table — The numbers of parous females allow the calculation of the mean durations of gonotrophic cycles and the percentage of parity according to time at each temperature. These values are used in Figs 2A and2B. (PDF) [file pone.0135489.s004.pdf]

24°C	Days	Nb of parous female x CG duration	Nomber of parous females	Cumulative number	Rate/29	Rounded rates/29	Rate/81	Rounded rates/81
	7	42	6	6	0,206897	0,21	0,0740741	0,07
	8	72	9	15	0,517241	0,52	0,1851852	0,19
	9	90	10	25	0,862069	0,86	0,308642	0,31
	11	22	2	27	0,931034	0,93	0,3333333	0,33
	12	12	1	28	0,965517	0,97	0,345679	0,35
	21	21	1	29	1	1	0,3580247	0,36
	Mean GC duration	**8,93**						

27°C	Days		Nomber of parous females	Cumulative number	Rate/60	Rounded rates/60	Rate/85	Rounded rates/85
	3	3	1	1	0,016667	0,02	0,0117647	0,01
	4	24	6	7	0,116667	0,12	0,0823529	0,08
	5	75	15	22	0,366667	0,37	0,2588235	0,26
	6	54	9	31	0,516667	0,52	0,3647059	0,36
	7	63	9	40	0,666667	0,67	0,4705882	0,47
	8	72	9	49	0,816667	0,82	0,5764706	0,58
	9	9	1	50	0,833333	0,83	0,5882353	0,59
	10	10	1	51	0,85	0,85	0,6	0,6
	12	12	1	52	0,866667	0,87	0,6117647	0,61
	13	26	2	54	0,9	0,9	0,6352941	0,64
	14	28	2	56	0,933333	0,93	0,6588235	0,66
	16	32	2	58	0,966667	0,97	0,6823529	0,68
	20	20	1	59	0,983333	0,98	0,6941176	0,69
	22	22	1	60	1	1	0,7058824	0,71
	Mean GC duration	**7,50**						

30°C	Days		Nomber of parous females	Cumulative number	Rate/61	Rounded rates/61	Rate/95	Rounded rates/95
	4	44	11	11	0,180328	0,18	0,1157895	0,12
	5	195	39	50	0,819672	0,82	0,5263158	0,53
	6	24	4	54	0,885246	0,89	0,5684211	0,57
	7	35	5	59	0,967213	0,97	0,6210526	0,62
	8	8	1	60	0,983607	0,98	0,6315789	0,63
	10	10	1	61	1	1	0,6421053	0,64
	Mean GC duration	**5,18**						
