# Supplementary material for: Parity and Longevity of Aedes aegypti According to Temperatures in Controlled Conditions and Consequences on Dengue Transmission Risks
Source: PLoS One. 2015 Aug 10;10(8):e0135489. doi: 10.1371/journal.pone.0135489 (PMC4530937; doi:10.1371/journal.pone.0135489)
Supplement: S2 Table — These data allow the calculation of the mean number of gonotrophic cycle, with their standard deviation, the mean duration of the all gonotrophic cycles, as well as the mean duration of the first, second and third gonotrophic cycle with their respective standard deviation, at each temperature. These values are used in Figs 3 and 4. (PDF) [file pone.0135489.s005.pdf]

Temperature	24°C																												
Female	1	2	3	4	5	6	7	8	9	10	11	12	13	14	15	16	17	18	19	20	21	22	23	24	25	26	27	28	29
Days of egg-laying after blood meal - Bold bars report another blood meal	8	9	9	8	7	8	7	7	12	9	9	8	9	9	8	8	9	9	7	11	8	8	7	9	21	9	7	8	11
	9	7	13	9	8	9	9	9	15	10	3		10	11		15	10	10		14	9		8	10		13	9	9	13
	12		19	8	9	12	11	11	19	11	7		12	12		19	13	12		15			10	13					15
					12	13	8						5	5			20						11	16					
						8								6									17	19					
						9								11															
						10																							
Number of GC	1	2	1	2	1	2	2	1	1	1	2	1	2	2	1	1	1	1	1	1	1	1	2	1	1	1	1	1	1
Duration of GC1	8	9	9	8	7	8	7	7	12	9	9	8	9	9	8	8	9	9	7	11	8	8	7	9	21	9	7	8	11
Duration of GC2		7		8		8	8				3		5	5									8						

Temperature	27°C																													
Female	1	2	3	4	5	6	7	8	9	10	11	12	13	14	15	16	17	18	19	20	21	22	23	24	25	26	27	28	29	30
Days of egg-laying after blood meal - Bold bars report another blood meal	8	6	4	7	8	13	7	8	14	16	5	8	5	8	9	5	22	7	7	20	13	14	8	5	5	6	5	4	5	5
	9	11	5	8			8	14	18		8		7	9	10	6		12	16	3	17	15	9	10	8	9		6	2	12
	9	12	6	9			3				11		8	11	12	8		13	17				13	11	9	11		9		13
			7	11							13		10		13	9		5					14	12	6	10		11		15
			4								5		11		7	11							5	13		11				
			5										14			13														
			8													16														
			10																											
			11																											
Number of GC	2	1	3	1	1	1	2	1	1	1	2	1	2	1	2	1	1	2	1	2	1	1	2	2	3	2	1	2	2	1
Duration of GC1	8	6	4	7	8	13	7	8	14	16	5	8	5	8	9	5	22	7	7	20	13	14	8	5	5	6	5	4	5	5
Duration of GC2	9		4				3				5		7		7			5		3			5	10	8	10		6	2	
Duration of GC3			8																						6					

Female	31	32	33	34	35	36	37	38	39	40	41	42	43	44	45	46	47	48	49	50	51	52	53	54	55	56	57	58	59	60
Days of egg-laying after blood meal - Bold bars report another blood meal	5	3	7	8	12	6	10	7	5	7	5	6	5	5	5	6	6	6	6	4	6	8	16	5	7	4	4	4	7	8
	7	6	8					15	9	11	6	7	9	9	7	11	8	4	9	6	9			6	6	6	5	5	9	11
	8		9						10	7	6				8	14	10	12	11	6	24			7	7	6	9	9	10	
	11								15	9	8				10	17			19	7					6	10		14	11	
	4								16						11	19				10										
	5								18							20														
																22														
Number of GC	2	2	2	1	1	1	1	1	2	2	3	1	1	1	2	1	2	2	1	3	1	1	1	1	3	3	2	1	1	1
Duration of GC1	5	3	7	8	12	6	10	7	5	7	5	6	5	5	5	6	6	6	6	4	6	8	16	5	7	4	4	4	7	8
Duration of GC2	4	6	8						9	7	6				8		8	4		6					6	6	9			
Duration of GC3											6									7					6	6				

Temperature	30°C																													
Female	1	2	3	4	5	6	7	8	9	10	11	12	13	14	15	16	17	18	19	20	21	22	23	24	25	26	27	28	29	30
Days of egg-laying after blood meal - Bold bars report another blood meal	4	7	5	5	4	5	5	5	7	5	5	5	5	4	4	4	5	6	5	5	5	5	7	4	10	4	5	5	4	5
			4	6	5		9	4		6	6	7	8	4	4		5			7	4	6				6	6			7
				4			11			7	7		9		4		8			5		7					4			
											8									7										
											3									8										
											4																			
Number of GC	1	1	2	2	1	1	1	2	1	1	2	1	1	2	3	1	2	1	1	2	2	1	1	1	1	1	2	1	1	1
Duration of GC1	4	7	5	5	4	5	5	5	7	5	5	5	5	4	4	4	5	6	5	5	5	5	7	4	10	4	5	5	4	5
Duration of GC2			4	4				4			3			4	4		5			5	4						4			
Duration of GC3															4															

Female	31	32	33	34	35	36	37	38	39	40	41	42	43	44	45	46	47	48	49	50	51	52	53	54	55	56	57	58	59	60	61
Days of egg-laying after blood meal - Bold bars report another blood meal	5	5	5	5	5	4	5	5	5	7	5	5	6	4	5	7	5	5	5	5	5	5	5	5	6	6	5	4	5	5	8
		8		7	7	5	4	6			8	8	4	4	6		6	6	4			4	9	4	5			6		4	5
		10			4	4					7		6				4								8						6
		11			8								8				6								9						10
																	8														
																	9														
																	2														
Number of GC	1	2	1	1	2	2	2	1	1	1	2	1	2	2	1	1	3	1	2	1	1	2	1	2	2	1	1	1	1	2	2
Duration of GC1	5	5	5	5	5	4	5	5	5	7	5	5	6	4	5	7	5	5	5	5	5	5	5	5	6	6	5	4	5	5	8
Duration of GC2		8			4	4	4				7		4	4			4		4			4		4	5					4	5
Duration of GC3																	2														
