# Supplementary material for: Parity and Longevity of Aedes aegypti According to Temperatures in Controlled Conditions and Consequences on Dengue Transmission Risks
Source: PLoS One. 2015 Aug 10;10(8):e0135489. doi: 10.1371/journal.pone.0135489 (PMC4530937; doi:10.1371/journal.pone.0135489)
Supplement: S4 Table — These data allow the calculation of survival rates, mortality rates, duration of life and daily probability of survival. These data are used in the modeling of daily probability of survival according to the age of the females at each experimental temperatures. These values are used in Figs 6 and 7. (PDF) [file pone.0135489.s007.pdf]

24°C (Model)

Days of study	Age of females	Nomber of alive	Number of dead	Survival (s)	Mortality	D*B	Life duration	p	Age	p
1	9	67	2	1,00	0,00	17,470	22,1827	0,9559	1,00	0,935
2	10	65	5	0,97	0,03	48,675	22,5965	0,9567	2,00	0,942
3	11	60	9	0,90	0,10	96,614	23,6683	0,9586	4,00	0,950
4	12	51	3	0,76	0,24	35,205	25,9506	0,9622	6,00	0,955
5	13	48	4	0,72	0,28	50,940	26,8391	0,9634	8,00	0,958
6	14	44	5	0,66	0,34	68,675	28,1213	0,9651	10,00	0,960
7	15	39	1	0,58	0,42	14,735	29,9657	0,9672	12,00	0,962
8	16	38	1	0,57	0,43	15,735	30,3665	0,9676	14,00	0,964
9	17	37	0	0,55	0,45	0,000	30,7620	0,9680	16,00	0,965
10	18	37	1	0,55	0,45	17,735	30,7620	0,9680	18,00	0,967
11	19	36	1	0,54	0,46	18,735	31,1238	0,9684	20,00	0,968
12	20	35	0	0,52	0,48	0,000	31,4778	0,9687	22,00	0,969
13	21	35	1	0,52	0,48	20,735	31,4778	0,9687	24,00	0,970
14	22	34	0	0,51	0,49	0,000	31,7938	0,9690	26,00	0,971
15	23	34	1	0,51	0,49	22,735	31,7938	0,9690	28,00	0,971
16	24	33	1	0,49	0,51	23,735	32,0683	0,9693	30,00	0,972
17	25	32	1	0,48	0,52	24,735	32,3287	0,9695	32,00	0,973
18	26	31	4	0,46	0,54	102,940	32,5736	0,9698	34,00	0,974
19	27	27	1	0,40	0,60	26,735	33,5868	0,9707	36,00	0,974
20	28	26	6	0,39	0,61	166,410	33,8503	0,9709	38,00	0,975
21	29	20	0	0,30	0,70	0,000	35,6849	0,9724	40,00	0,975
22	30	20	1	0,30	0,70	29,735	35,6849	0,9724	42,00	0,976
23	31	19	0	0,28	0,72	0,000	35,9981	0,9726	44,00	0,976
24	32	19	1	0,28	0,72	31,735	35,9981	0,9726	46,00	0,977
25	33	18	1	0,27	0,73	32,735	36,2349	0,9728	48,00	0,977
26	34	17	0	0,25	0,75	0,000	36,4408	0,9729	50,00	0,978
27	35	17	2	0,25	0,75	69,470	36,4408	0,9729	52,00	0,978
28	36	15	1	0,22	0,78	35,735	36,6683	0,9731	54,00	0,979
29	37	14	14	0,21	0,79	514,289	36,7349	0,9731	56,00	0,979
30	51	0	0	0,00	1,00	0,000				
31	**Age moyen des femelles à j0**					**Durée de vie**	**31,70**			
32	**7,73**					**Moyenne**	18,26	0,9467		

27°C (Model)

Days of study	Age of females	Nomber of alive	Number of dead	Survival (s)	Mortality	D*B	Life duration	p	Age	p
1	14	85	4	1,00	0,00	57,365	32,7882	0,9700	1,00	0,961
2	15	81	5	0,95	0,05	76,706	33,6992	0,9708	2,00	0,964
3	16	76	0	0,89	0,11	0,000	34,9070	0,9718	4,00	0,966
4	17	76	3	0,89	0,11	52,024	34,9070	0,9718	6,00	0,968
5	18	73	0	0,86	0,14	0,000	35,6288	0,9723	8,00	0,969
6	19	73	0	0,86	0,14	0,000	35,6288	0,9723	10,00	0,970
7	20	73	1	0,86	0,14	20,341	35,6288	0,9723	12,00	0,970
8	21	72	1	0,85	0,15	21,341	35,8412	0,9725	14,00	0,971
9	22	71	0	0,84	0,16	0,000	36,0454	0,9726	16,00	0,971
10	23	71	0	0,84	0,16	0,000	36,0454	0,9726	18,00	0,972
11	24	71	0	0,84	0,16	0,000	36,0454	0,9726	20,00	0,972
12	25	71	4	0,84	0,16	101,365	36,0454	0,9726	22,00	0,973
13	26	67	3	0,79	0,21	79,024	36,6845	0,9731	24,00	0,973
14	27	64	0	0,75	0,25	0,000	37,1693	0,9735	26,00	0,973
15	28	64	2	0,75	0,25	56,682	37,1693	0,9735	28,00	0,974
16	29	62	2	0,73	0,27	58,682	37,4541	0,9737	30,00	0,974
17	30	60	0	0,71	0,29	0,000	37,7245	0,9738	32,00	0,974
18	31	60	1	0,71	0,29	31,341	37,7245	0,9738	34,00	0,974
19	32	59	1	0,69	0,31	32,341	37,8327	0,9739	36,00	0,974
20	33	58	1	0,68	0,32	33,341	37,9274	0,9740	38,00	0,975
21	34	57	1	0,67	0,33	34,341	38,0078	0,9740	40,00	0,975
22	35	56	2	0,66	0,34	70,682	38,0733	0,9741	42,00	0,975
23	36	54	3	0,64	0,36	109,024	38,1745	0,9741	44,00	0,975
24	37	51	3	0,60	0,40	112,024	38,2824	0,9742	46,00	0,975
25	38	48	48	0,56	0,44	1840,376	38,3412	0,9743	48,00	0,976
26	56	0	0	0,00	1,00				50,00	0,976
27									52,00	0,976
28									54,00	0,976
29									56,00	0,976
30										
31	**Age moyen des femelles à j0**					**Durée de vie**	**36,55**			
32	**13,34**					**Moyenne**				

30°C (Model)

Days of study	Age of females	Nomber of alive	Number of dead	Survival (s)	Mortality	D*B	Life duration	p	Age	p
1	19	95	1	1,00	0,00	18,874	27,5158	0,9643	1,00	0,913
2	20	94	4	0,99	0,01	79,495	27,6077	0,9644	2,00	0,925
3	21	90	4	0,95	0,05	83,495	27,9515	0,9649	4,00	0,937
4	22	86	5	0,91	0,09	109,368	28,2807	0,9653	6,00	0,944
5	23	81	15	0,85	0,15	343,105	28,6762	0,9657	8,00	0,949
6	24	66	6	0,69	0,31	143,242	29,9949	0,9672	10,00	0,952
7	25	60	7	0,63	0,37	174,116	30,6070	0,9679	12,00	0,956
8	26	53	9	0,56	0,44	232,863	31,3643	0,9686	14,00	0,958
9	27	44	2	0,46	0,54	53,747	32,4873	0,9697	16,00	0,961
10	28	42	13	0,44	0,56	362,358	32,7546	0,9699	18,00	0,963
11	29	29	3	0,31	0,69	86,621	34,9426	0,9718	20,00	0,964
12	30	26	4	0,27	0,73	119,495	35,6429	0,9723	22,00	0,966
13	31	22	1	0,23	0,77	30,874	36,6919	0,9731	24,00	0,968
14	32	21	2	0,22	0,78	63,747	36,9689	0,9733	26,00	0,969
15	33	19	3	0,20	0,80	98,621	37,5053	0,9737	28,00	0,970
16	34	16	1	0,17	0,83	33,874	38,3737	0,9743	30,00	0,971
17	35	15	0	0,16	0,84	0,000	38,6737	0,9745	32,00	0,973
18	36	15	1	0,16	0,84	35,874	38,6737	0,9745	34,00	0,974
19	37	14	0	0,15	0,85	0,000	38,8737	0,9746	36,00	0,975
20	38	14	0	0,15	0,85	0,000	38,8737	0,9746	38,00	0,976
21	39	14	14	0,15	0,85	544,232	38,8737	0,9746	40,00	0,976
22	40	0	0	0,00	1,00				42,00	0,977
23									44,00	0,978
24									46,00	0,979
25									48,00	0,980
26									50,00	0,980
27									52,00	0,981
28									54,00	0,982
29									56,00	0,982
30										
31		**Age moyen des femelles à j0**				**Durée de vie**	**7,49**			
32		**17,87**				**Moyenne**				

24°C

jours du suivi	Age femelles	nbre de fem pares	Nbre cumulé	Taux de parité	P	Life Expectation	Expectation of infective life	
1	2	0	0	0,00	0,942	16,862	0,8	16,012
2	3	0	0	0,00	0,947	18,321	2,3	
3	4	0	0	0,00	0,950	19,513	3,5	
4	5	0	0	0,00	0,953	20,548	4,5	
5	6	0	0	0,00	0,955	21,476	5,5	
6	7	0	0	0,00	0,956	22,328	6,3	
7	8	6	6	0,07	0,958	23,120	7,1	
8	9	9	15	0,19	0,959	23,866	7,9	
9	10	10	25	0,31	0,960	24,575	8,6	
10	11	0	25	0,31	0,961	25,252	9,2	
11	12	2	27	0,33	0,962	25,903	9,9	
12	13	1	28	0,35	0,963	26,532	10,5	
13	14	0	28	0,35	0,964	27,141	11,1	
14	15	0	28	0,35	0,965	27,734	11,7	
15	16	0	28	0,35	0,965	28,311	12,3	
16	17	0	28	0,35	0,966	28,876	12,9	
17	18	0	28	0,35	0,967	29,429	13,4	
18	19	0	28	0,35	0,967	29,972	14,0	
19	20	0	28	0,35	0,968	30,505	14,5	
20	21	0	28	0,35	0,968	31,030	15,0	
21	22	1	29	0,36	0,969	31,547	15,5	

27°C

jours du suivi	Age femelles	nbre de fem pares	Nbre cumulé	Taux de parité	P	Life Expectation	Expectation of infective life	
1	2	0	0	0,00	0,964	27,091	16,4	10,670
2	3	0	0	0,00	0,965	28,283	17,6	
3	4	1	1	0,01	0,966	29,194	18,5	
4	5	6	7	0,08	0,967	29,940	19,3	
5	6	15	22	0,26	0,968	30,578	19,9	
6	7	9	31	0,36	0,968	31,140	20,5	
7	8	9	40	0,47	0,969	31,642	21,0	
8	9	9	49	0,58	0,969	32,099	21,4	
9	10	1	50	0,59	0,970	32,519	21,8	
10	11	1	51	0,60	0,970	32,908	22,2	
11	12	0	51	0,60	0,970	33,271	22,6	
12	13	1	52	0,61	0,971	33,612	22,9	
13	14	2	54	0,64	0,971	33,935	23,3	
14	15	2	56	0,66	0,971	34,240	23,6	
15	16	0	56	0,66	0,971	34,531	23,9	
16	17	2	58	0,68	0,972	34,808	24,1	
17	18	0	58	0,68	0,972	35,074	24,4	
18	19	0	58	0,68	0,972	35,329	24,7	
19	20	0	58	0,68	0,972	35,574	24,9	
20	21	1	59	0,69	0,972	35,811	25,1	
21	22	0	59	0,69	0,973	36,039	25,4	

30°C

jours du suivi	Age femelles	nbre de fem pares	Nbre cumulé	Taux de parité	P	Life Expectation	Expectation of infective life	
1	2	0	0	0,00	0,925	12,753	6,0	6,769
2	3	0	0	0,00	0,932	14,114	7,3	
3	4	1	1	0,01	0,937	15,262	8,5	
4	5	6	7	0,07	0,940	16,284	9,5	
5	6	15	22	0,23	0,944	17,223	10,5	
6	7	9	31	0,33	0,946	18,103	11,3	
7	8	9	40	0,42	0,949	18,939	12,2	
8	9	9	49	0,52	0,951	19,742	13,0	
9	10	1	50	0,53	0,952	20,518	13,7	
10	11	1	51	0,54	0,954	21,273	14,5	
11	12	0	51	0,54	0,956	22,011	15,2	
12	13	1	52	0,55	0,957	22,736	16,0	
13	14	2	54	0,57	0,958	23,449	16,7	
14	15	2	56	0,59	0,959	24,155	17,4	
15	16	0	56	0,59	0,961	24,853	18,1	
16	17	2	58	0,61	0,962	25,546	18,8	
17	18	0	58	0,61	0,963	26,235	19,5	
18	19	0	58	0,61	0,964	26,921	20,2	
19	20	0	58	0,61	0,964	27,605	20,8	
20	21	1	59	0,62	0,965	28,288	21,5	
21	22	0	59	0,62	0,966	28,971	22,2	

24°C Model | 27°C Model | 30°C Model
