# Supplementary material for: Parity and Longevity of Aedes aegypti According to Temperatures in Controlled Conditions and Consequences on Dengue Transmission Risks
Source: PLoS One. 2015 Aug 10;10(8):e0135489. doi: 10.1371/journal.pone.0135489 (PMC4530937; doi:10.1371/journal.pone.0135489)
Supplement: S5 Table — The percentage of female potentially able to transmit the virus increases from 28% at 24°C to 43% and 39% at 27°C and 30°C respectively. (PDF) [file pone.0135489.s008.pdf]

	Temperatures (°C)		
	**24**	**27**	**30**
Females taking 1 blood-meal (%)	60%	86%	96%
Females taking 2 blood-meals and having 2 GC* (%)	28%	43%	39%
potentially able to transmit the virus (%)	28,0%	43,0%	39,0%

* GC = Gonotrophic Cycle
